# Socioeconomic and religious differentials in contraceptive uptake in western Ethiopia: a mixed-methods phenomenological study

**DOI:** 10.1186/s12905-018-0580-6

**Published:** 2018-06-05

**Authors:** Setegn Tigabu, Tesfa Demelew, Awol Seid, Bisrate Sime, Tsegahun Manyazewal

**Affiliations:** Ethiopian Public Health Association, P.O. Box 7117, Addis Ababa, Ethiopia

**Keywords:** Family planning, Contraceptive, Quality, Socioeconomic, Religion, Ethiopia

## Abstract

**Background:**

Despite the large-scale investment in access to contraceptives, high population growth and unintended pregnancies are posing pressures in Ethiopia where the economy is incapable of holding overpopulation. The aim of this study was to assess and explore socioeconomic and religious differentials in contraceptive uptake.

**Methods:**

A mixed-methods phenomenological study was conducted in western Ethiopia, Oromia region. Data were collected through survey with 1352 mothers of reproductive age, interviews with 37 key informants, and 13 focus group discussions with family planning service providers, college instructors and mothers of reproductive age. Multivariate logistic regression model was used to identify factors associated with contraceptives uptake and thematic analysis was used to interpret the qualitative data.

**Results:**

Of mothers included, 68% lived in rural settings and 50% were unable to read and write. Religiously, 42% were Protestant Christian, 30% Orthodox Christian and 25% Muslim. Modern-contraceptives were available at healthcare facilities; however, all mothers have been influenced by religion not to use contraceptives. Muslims were 65% less likely to utilize modern-contraceptives as compared to Orthodox (a*OR*, .35, 95% CI, .21–.60). All mothers were well informed of any one of modern-contraceptive methods and knew a place to get the service, while their knowledge about contraceptive was limited and their contraceptive uptake was low.

**Conclusion:**

Though the Ethiopian government has so far improved access to contraceptives, utilization is lagging, mainly due to religious influences, limited contraceptives knowledge in the community, and low home-based contraceptive coverage. Societal attitudes and norms of the community towards modern-contraceptives need to be modified through innovative and culturally appropriate interventions. In countries like Ethiopia, where people’s religious devotion remains reasonably high, knowledge on natural-contraceptive methods is equally important to help religious people make an informed decision about family planning in accordance with their faith.

**Electronic supplementary material:**

The online version of this article (10.1186/s12905-018-0580-6) contains supplementary material, which is available to authorized users.

## Background

Over the past five decades, Ethiopia has been proactively responding to the Global call for accelerated efforts meant for increasing access to and utilization of quality family planning services that can empower people to make informed choices about the number and spacing of their children. The country has been working intensely with other committed countries to meet the global family planning 2020 (FP2020) targets aimed at expanding access to contraceptives. Reports have testified Ethiopia’s commitment towards increasing family planning related budgets [1]. According to the Central Statistical Agency of Ethiopia, the use of modern family planning methods in the country increased considerably with an upward trend of contraceptive prevalence rate from 8% in 2000 to 42% in 2015 [2].

Despite all these efforts have made and specific progresses counted, Ethiopia is still behind the global family planning goals and targets. The total fertility rate in the country is high at 4.5 and that of adolescent birth rate is high at 63 per 1000 [2]. Family planning service indicators lack standardisations to serve as the basis for comparison at different levels of the health delivery system. Besides, the link between household and primary healthcare units has not been tied enough to deliver ideal contraceptive services.

Studies conducted in some countries reported that socioeconomic status [3–7] and religion [8–12] influence contraceptive use rate of mothers in reproductive age, while this has been explored less in Ethiopia. In some religions, children are looked as a blessing and a gift from Almighty (God/Allah) and it is sinful to prevent pregnancies. In these religions, each sexual act in a marriage needs to be open to the possibility of conceiving a child, despite an artificial contraception which is contrary to Almighty’s will for marriage because it separates the act of conception from sexual union. While some other religions permit contraception at least in the concept of healthy timing and spacing of pregnancy. Both conditions, however, are channelled to communities through religious leaders who would influence decisions around contraceptives negatively or positively. With regards to socioeconomic status, some studies considered limited awareness and/or knowledge, misconceptions, poor partner communication, fear of side effects, and inaccessibility of contraceptives as the major barriers to contraceptive uptake [13–15].

The Ministry of Health of the Federal Democratic Republic of Ethiopia (FMoH) has collaborated with different national and international implementing partners to improve contraceptive uptake in Ethiopia. By 2020, the country plans to increase modern contraceptive prevalence rate from 32 to 40% for women ages 15 to 19 years and from 38 to 43% for those women ages 20 to 24 years; reduce unmet need for family planning from 20 to 10% for women ages 15 to 19 years and from 18 to 10% for those women ages 20 to 24 years; and reduce adolescent pregnancy rate from 12 to 3% [16]. Translating the plans to action requires a deeper understanding of existing and emerging challenges detracting contraceptive uptake at community and at various health sectors levels. Thus, the aim of this study was to assess and explore the socioeconomic and religious differentials in contraceptive uptake.

## Methods

A mixed-method, cross-sectional phenomenological study was conducted at household, health facilities, health bureaus, and training institutions’ levels in Western Ethiopia, Oromia region. Oromia Region was selected as it the largest and heavily populated region in Ethiopia [17] with diverse religion, culture and socioeconomic status.

### Household survey

The household survey was conducted in five zones of Oromia Region; namely, West Shewa, West Wellega, East Wellega, Elluababora, and Jimma zones. West Shewa is located 126 Km away from central Ethiopia. The zone had 19 districts, an estimated total population of the 2,395,843, women in reproductive age group of 529,481, and women eligible for family planning service of 441,074. West Wollega zone is located 441 km away from central Ethiopia. The zone had 21 districts and an estimated total population of 1,655,954, women in reproductive age group of 366,463, and women eligible for family planning service of 304,696. East Wellega zone is located 325 Km away from central Ethiopia. The zone had 17 districts and an estimated total population of 1,383,036. Elluababora zone is located 650 Km away from central Ethiopia. The zone had 24 districts, an estimated total population of 1,545,820, women in reproductive age group of 284,431, and women eligible for family planning service of 230,389. Jimma zone is located 350 Km away from central Ethiopia. The zone had 18 districts and an estimated total population of 3,030,740.

The study population of the household survey was all married women in reproductive age. Multistage sampling technique was employed to select study participants. Of the total 99 districts in the five zones, eight districts - one from Jimma zone, one from East Wollega and two from each of the other three zones - were selected randomly using proportional allocation and considering logistics, time and geographical accessibility. Two kebeles (the lowest administrative unit in Ethiopia) were selected from each district randomly using equal allocation. Stratifying the kebele’s into urban and rural, 16 kebeles (three from urban and 13 from rural) were included. From these sites, a total of 1354 mothers of reproductive age were selected using single population proportion formula and considering a design effect of 1.5 and a non-response rate of 10%. The inclusion criteria was married women in reproductive age (15–49 years), while the exclusion criteria were women who were not married, not a permanent resident in the kebele (live in the kebele less than 6 months), or unable to answer survey questions due to health problems or severe illnesses.

The data collection tool was a semi-structured, pre-tested interviewer-administrated questionnaire adapted from a range of Demographic and Health Survey (DHS) tools [18]. The questionnaire captured socio-demographic variables, reproductive and pregnancy history, fertility preferences, and knowledge about and utilization of family planning services (Additional file 1).

### Facility-based survey

The facility-based survey included district health offices, health centers, health posts, and midwife/nurse training institutions in the 99 districts. Data were collected through focus group discussions and in-depth interviews. The focus group discussion was conducted with purposively selected three groups, which included family planning service providers, college instructors, and women eligible for contraceptives. The in-depth interview included 37 key informants including health extension workers, head of healthcare facilities, head of zonal and district health offices, and midwifery/nursing training college/school deans selected with purposive sampling. Guiding questions of the qualitative studies focus on quality of contraceptive services, quality of teaching-learning at midwifery/nursing training institutions, and barriers to utilization of family planning services. Qualitative data were recorded using a tape recorder and notes were taken after getting consent from the participants. Interviews were conducted until a saturation point was reached.

### Study variables

The independent variables were socioeconomic, religious, and socio-demographic factors while the dependent variable was a current use of contraceptives.

### Operational definitions

Operational definitions of major variables used in this study are as follows:

*Contraceptive prevalence rate*- refers to the proportion of currently married women of reproductive age who are using a contraceptive method at the time of the study.

*Current use of contraception* (met need) - current use of any modern-contraceptive methods.

*Contraceptive knowledge*- derived from contraceptive knowledge specific questions, where a respondent is determined knowledgeable, fairly knowledgeable, or not knowledgeable if scored greater or equal to 75%, 50–75%, or less than 50%, respectively.

*Ever use of contraception*- refers to any history of using a particular contraceptive method.

*Choice of methods* (Method mix) – refers the relative level of use of different contraceptive among those available.

*Informed choice* – refers to a decision that a woman can select the contraceptive method that best satisfies her personal, reproductive and health, based on substantial information and a range of contraceptive options.

*Sources of modern contraceptive methods* – refers to the place where a woman obtained the current contraceptive and the means from which she heard about contraceptives.

*Contraceptive discontinuation* - refers to stopping the use of the current contraceptive method for various reasons.

*Decision-making on seeking contraceptive use* – refers to the decision making process to seek and use contraception, which could be made mainly by the respondent herself, the husband or partner, as a joint decision between the two, or by some other person.

### Data processing and analysis

The association between potential factors and current use of contraceptives was assessed using multivariate logistic regression model. A thematic content analysis approach focusing on the study questions was used for qualitative analysis of data from notes, interview forms, and records and quantitative and qualitative results triangulated. Data quality was assured through using adapted standard data collection instruments, conducting pre-testing for possible modifications, training of data collectors and pre-data collection idea exchange, enhancing privacy of respondents, and close supervision of data collection procedures.

Ethical clearance was obtained from the Internal Scientific and Review Committee of the Ethiopian Public Health Association and permissions to conduct the research were obtained from regional, Zonal, and district health bureaus and training institutions. The ethical committee approved a verbal consent procedure involving informed consent process as the study had minimal risk to respondents and only involved procedures for which consent is not normally sought. Data collectors provided respondents with a written statement about the study and obtained their verbal consent. The data collectors signed on the consent to document each respondent’s consent, while the investigators kept the consents separately from filled questionnaires. For married women participants below the age of 18, in addition to their assents, verbal consent to participate was obtained from their husband (if above 18 years of age) or parents. All necessary measures were taken to maintain confidentiality.

## Results

### Quantitative findings

#### Socio-demographic characteristics of the respondents

A total of 1352 married women replied to the questionnaire. Fifty-three percent were in the age range 25 to 34; 68% lived in rural settings; 45% were housewives; 33.5% were farmers; and 51% have never gone to school and were unable to read and write. Religiously, 42% of percent respondents followed Protestant Christian religion, followed by Orthodox Christian (30%) and Muslim (25%) (Table 1).

**Table 1:** Socio-demographic characteristics of married women participated in the study (*n* = 1352)

Variables		Number	Percent
Residence	Urban	433	32.0
	Rural	919	68.0
Age	15–24	299	22.2
	25–34	710	52.5
	35–49	343	25.3
Level of Education	No schooling/unable to read & write	687	50.8
	No schooling but read and write	22	1.6
	Primary, Secondary and Preparatory School	609	45
	Diploma and above	34	2.5
Partner’s Educational level	No schooling/unable to read & write	343	25.4
	No schooling but read and write	64	4.7
	Primary, Secondary or Preparatory School	848	62.7
	Diploma and above	82	6.1
	Others	15	1.1
Religion	Orthodox	406	30.
	Muslim	343	25.4
	Catholic	2	0.1
	Protestant	563	41.6
	Other (Hawariat, Wakefeta)	38	2.8
Occupation	Farmer	454	33.5
	Business man (merchant)	197	14.5
	Government employee	39	2.9
	Private employee	20	1.5
	Housewife	614	45.3
	Daily laborer	25	1.8
	Others (Student, …)	3	0.2

### Reproductive and abortion history

Of the total 1352 married women participated in the study, 96.8% had ever been pregnant at least once in their life. The frequency of their pregnancies ranged from 1 to 13, of which 99.5% had given birth at least once and 10% of these had given birth 8 times or more. Abortion and still birth had occurred in 14.4% and 13.5% of participants, respectively (Table 2).

**Table 2:** Reproductive and abortion history of respondents

Variables	Number	Percent
Ever been pregnant (*n* = 1352)		
No	43	3.2
Yes	1309	96.8
Frequency of Pregnancy (*n* = 1297)		
1	194	15.0
2–4	612	47.2
5–7	363	28.0
8–13	128	9.9
Ever given birth (*n* = 1304)		
No	6	0.5
Yes	1298	99.5
Still Birth (n = 1304)		
No	1127	86.5
Yes	176	13.5
Number of still births (*n* = 176)		
1–3	172	97.7
4–7	4	2.3
Women who have ever faced abortion		
No	1112	85.6
Yes	187	14.4

### Family size preferences and pregnancy preparedness

Fifty-one percent of respondents had 2 to 4 living children and 67.9% had one under 5 children, and 78.3% assumed that a family should have 2 to 4 children. Of the total respondent, 55% showed interest to have more children than they had, of whom 78% planned to give birth within the following two years (Table 3). For women who do not want to have additional child in the near future, their major reasons were absence of someone to take care of their children (48.2%), as they recently gave birth (19.5%), and due to limited income (17.1%). Sixty two percent of the women had joint decision with their husbands on the number of children they should have.

**Table 3:** Family size preference and pregnancy preparedness of respondents

Variables	Number	percent
Number of alive children (*n* = 1301)		
1	224	16.6
2–4	658	50.7
5–7	339	26.1
8–12	85	6.6
Number of < 5 children (*n* = 1266)		
0	35	2.8
1	859	67.9
2–5	372	29.4
Number of children family should have (*n* = 1301)		
1	38	2.9
2–4	1019	78.3
5–10	244	18.8
Like to have more children (*n* = 1330)		
No	577	43.4
Yes	730	54.8
Do not know	23	1.7
Numbers of more children like to have (*n* = 675)		
1	259	34.8
2	253	34.0
3+	163	22.0
Plan to have another birth (*n* = 736)		
Within 2 years	60	8.0
After 2 years	584	77.5
Do not know	92	12.2
Number of child partner want (*n* = 1254)		
Both want same number	844	62.7
Husband want more children	285	21.2
Husband want fewer children	125	9.3

### Knowledge of contraceptives and sources of information

Almost all respondents (98%) had heard of any one of contraceptive methods, particularly about injectables (99%), pills (94%), implants (88%), and Intrauterine Contraceptive Devices (IUCDs) (48%). In addition, 98% of them knew a place where they could get contraceptive services. However, only 43% were knowledgeable about contraceptives. Health extension workers played the leading role (75%) in information dissemination of contraceptive methods. Among public health facilities, 79, 54 and 17% of mothers stated health centers, health posts, and private clinics, respectively, as facilities where they could get contraceptive services.

### Current and ever use of contraceptives

Seventy-one percent of women reported ever use of contraceptives, of whom 76% were Protestant, 75% Orthodox, and 61% Muslim. Out of these, 36% used the contraceptives for limiting and 64% for spacing children (Table 4).

**Table 4:** Proportion of women who ever and currently uses contraceptives by socio-demographic characteristics

Socio demographic Characteristics		Ever contraceptive use		Current contraceptive use	
		Number	%	Number	%
Occupation	Farmer	273	62.6	205	47.1
	Business Woman	152	77.2	138	70.4
	Government employee	35	92.1	33	84.6
	Private employed	14	73.7	17	85.0
	House Wife	449	74.1	369	61.0
	Daily laborer	19	76.0	19	76.0
	Other	3	100.0	3	100.0
Age Group	15–19	35	62.5	36	64.3
	20–24	175	73.5	167	70.5
	25–29	312	76.3	256	62.4
	30–34	210	73.2	165	57.7
	35–39	163	69.4	115	48.9
	40–44	42	54.5	38	49.4
	45–49	7	36.8	6	31.6
Religion	Orthodox	295	74.5	251	63.5
	Muslim	205	61.4	154	46.1
	Catholic	2	100.0	2	100.0
	Protestant	422	76.0	364	65.6
	Other	20	55.6	13	36.1
Educational Level	No school /unable to read & write	424	63.8	334	50.4
	No school but read and write	14	63.6	11	50.0
	Primary school [grade1–8]	356	76.6	302	64.8
	Secondary school [grade 9–10]	103	88.0	94	81.0
	Preparatory school [grade 11–12]	20	90.9	13	59.1
	Diploma	23	88.5	23	88.5
	Degree and above	5	71.4	7	87.5

**Fig. 1:**
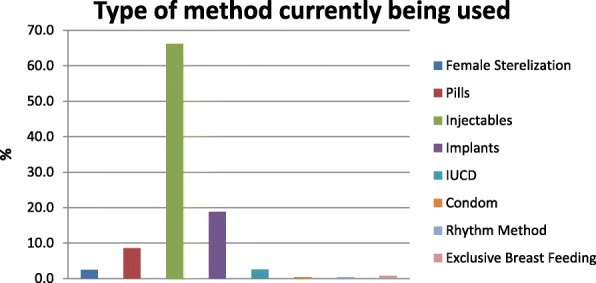
Types of contraceptive methods currently in use by women

The current use of modern-contraceptives was 59%. Injectables were the most commonly used ever (62.7% and current (66.2%) used methods, followed by implants (ever 5.1% and current 18.8%) and pills (ever 31.3% and current 8.5%) (Fig. 1). The decision to seek contraceptive was made jointly with husbands for majority (58%) of women, and one-fifth of the women were sole decision makers. About 64% recalled as they had been counseled about contraceptive methods and related issues by contraceptive service providers, while 10% were informed about method effectiveness and 3% were instructed how to use.

About half (52%) of mothers obtained their choice of contraceptives from health centers, 31% from health posts through health extension workers, and 3% from government or private hospitals and pharmacies. Very few complained on distance and accessibility of the facilities, costs of services, or optimism of facility workers. Besides, the health facilities rarely encountered contraceptive stock-outs or family or social resistance. However, during the previous 12 months, home-visit coverage of contraceptives was 23 and 51% of women did not visit health facilities for contraceptive services. Women in the age group 25–34 were 1.58 times more likely to utilize contraceptives as compared to those women in the age group of 15–24 (AOR = 1.58, 95% CI: 1.12, 2.24) (Table 5).

**Table 5:** Logistic regression analyses of the relative effect of socioeconomic, religion and socio-demographic variables on contraceptive uptake

Explanatory Variables	Contraceptive used		Crude OR(95%CI)	Adjusted OR (95% CI)	*p*-value
	Yes (n)	No (n)			
Age (years)					
15–24	210	84	1		
25–34	522	174	1.20 (.88, 1.63)	1.58((1.12, 2.24)	0.01
35–49	212	119	0.71 (0.51, 0.99)	1.14(0.77, 1.67)	0.5
Religion					
Orthodox	295	101	1		
Muslim	205	129	0.54(0.40, 0.75)***	0.35(0.21, 0.60)	0.000
Protestant + Catholic	444	149	1.02(0.76, 1.37)	0.95(0.66, 1.35)	0.76
Educational Background					
No School	794	358	1		
Primary School	103	14	3.31(1.87, 5.88) ***	2.09(1.11, 3.92)	0.02
Secondary School	48	7	3.09(1.39, 6.90) **	1.52(0.64, 3.61)	0.35
Partner’s Occupation					
Farmer	549	298	1		
Merchant	138	33	2.27(1.51, 3.40) ***	2.07(1.33, 3.25)	0.001
Employed (Government, Private, Daily laborer and others)	258	47	2.98(2.12, 4.19) ***	2.34(1.59, 3.46)	0.000
Zone					
East Wollega	124	90	1		
Illubabor	150	34	3.20(2.02, 5.08) ***	6.11(3.27, 11.42)	0.02
Jimma	101	80	0.92(0.61, 1.37)	2.06(1.17, 3.67)	0.01
West Showa	262	124	1.53(1.09, 2.17) *	1.38(0.93, 2.03)	0.11
West Wollega	308	51	4.38(2.93, 6.55) ***	4.92(3.22, 7.53)	0.000

***Significant at *p*-value < 0.001, **at *p*-value < 0.01, *at *p*-value < 0.05

All mothers had religious pressure not to use modern-contraceptives, irrespective of the type of religion. Those who were Orthodox Christians were 1.65 times more likely to utilize contraceptives as compared to Muslims or Muslims were 65% less likely to utilize the services as compared to Orthodox Christians (AOR = .35, 95% CI: .21, .60). The other categories of religion (Protestant, Catholic and others) did not show association. Women who had a primary and secondary or above level of education were 2.09 and 1.52 times more likely to utilize contraceptives, respectively, as compared to those who did not have the same level of education (AOR 2.09, 95% CI: 1.11, 3.92; 1.52, 95% CI: .64, 3.46) (Table 5). A woman whose partner or husband is merchant was 2.07 times more likely to utilize contraceptives (AOR =2.07, 95% CI: 1.33, 3.25) as compared to those woman whose partner’s or husband’s occupation was farming. Likewise a woman whose partner or husband was government employee, private employee or daily laborer was 2.34 times (AOR = 2.34, 95% CI: 1.59, 3.46) more likely to utilize contraceptives as compared to those woman whose partner’s or husband’s occupation was farming.

### Qualitative findings

According to most mother discussants, community awareness has been established to the level that everyone was able to protect unwanted pregnancies, mainly through health education sessions when they visited health centers. Most discussants had the savoir-faire on the advantages of taking contraceptives for saving wealth, growth of children, mother and children’s health, and overall well-being of families. Supporting this argument, a respondent woman stated, “*If we didn’t take contraception properly we might give birth now and then without reasonable spacing. As a result, we might not be in a position to breastfeed our child for at least six months and if that is not the case the child may not have good resistance to many diseases. Lack of breastfeeding also affects mental growth of the child.*”

It is observed that there was no scarcity of contraceptives in the study zones. However, sometimes ago, there was a scarcity of Depo-Provera shot (Dipo) and pills. One service provider stated, “*For a short period of time Dipo was not available not only in our health center but also at the district level. Severely, there was also a shortage of lidocane, glove, etc. We have been supplied by different organizations. IPAS (which played a major role in solving such acute shortages) has given us long-acting contraceptives, including consumables like alcohol, glove and cotton.*”

As has been ascertained by the key informants, most of the women preferred to use short-acting contraceptives, particularly Dipo, as it is believed to be suitable for them as compared to other long-acting methods. Even those who had a maximum number of children (eight or more) preferred Dipo for such reason.

There were guidelines and procedures to support family planning service deliveries. These documents recommend the need of counseling clients about contraceptive procedures at their first visit, then delivering the service accurately. In some health facilities, there were some challenges insisting health workers to breach guidelines. One discussant service provider mentioned: “*After we counsel, they tend to use their previous choice. One of my clients asked me whether I had Dipo to give her or not at the moment. If I fail to do so, she will go back to home and does not want to use anything other than that. She did not want to receive what I recommended.*”

There was also limitation on the skill of contraceptive service provision. One respondent said, “*My sister wants to use implants and goes to a health center. During the insertion, there was excessive bleeding and she suffered a lot. Within a very short period, she went to hospital for removal before the end of prevention period. Now she is using Dipo instead of implants”.*

Implanon and IUCD were not being highly utilized by the community. Major reasons mentioned were religious pressure, lack of skill of service delivery, unwillingness of husband/partners, feeling shameless to have the IUCD inserted in the uterus, and fear of side effects. For Muslim women in both rural and urban settings, religious pressure was the commonest reason. Supporting this argument, one health provider said, “*The main challenge our clients raised on long-acting family planning (LAFT) is related to religion. Muslims believe that if a mother happens to pass away with Implanon inserted, her soul would not get into heaven. We showed them all the methods available but they have no tolerance even to finish the counseling session. When they hear the name “implanon” they appear outraged*”.

The other reason for the low-level utilization of Implanon and IUCD was lack of skilled and trained health professionals to provide the services. As witnessed by a service provider in one of the health facilities, after counseling a mother to use IUCD for family planning, no one was around to provide the method.

There were also misconceptions and beliefs in the community about LAFP, mainly a fear of side effects. Some of the side effects did not exist in the real sense and some were too exaggerated. The common side effects mentioned by the respondents were that women using implants need additional food; could be exposed to psychosis; their body blood levels could decrease; could develop headache; or got painful arms, and the like. They believed contraceptives inserted in uterus cause infertility, and the ones implanted in the arms lead to faintness. On the other hand, there was a group in the community which perceived contraceptives were being used in order to limit the size of their ethnic population.

Health extension workers, most of the time, were capable of providing contraceptive services which do not require complicated clinical procedures like condoms, Pills and Dipo and usually refer to the health center those who want to use LAFP methods. According to one discussant, trained health extension workers were trying to provide Implanon. However, there was shortage of supply and they were forced to refer women demanding LAFP methods to nearby hospital.

There were zonal reports and data compiled for family planning services for the past five years. However, there was no formal trend analysis conducted to identify factors affecting reproductive health and family planning services utilization and to indicate quality of counseling and methods mix.

In the earlier days, there was an old curriculum used to train regular midwife students for three years. Currently, the college is conducting “accelerated midwifery training” for those primarily trained in nursing for one year. There was a difference between the two curricula in terms of reproductive health and family planning contents. According to the discussants, unlike the previous curriculum, reproductive health and family planning topics are fascinatingly included as a course in the new curriculum. In the colleges, recording books, tally sheets, and Implanon were available. However, family planning training materials were not sufficient. One of the Health Science Colleges dean said, “*We are taking some materials from the nearby hospital and there is a time when we cannot find any logistics and students complained about the situation and sometimes we use videos instead of demonstration*”. According to most discussants, there is skill gap among instructors on LAFP. Supporting this argument an instructor said, “*I have taken contraceptive learning as only one sub-topic. I am teaching midwifery based on the knowledge and skill I have gotten from that training. I do not know any instructor who was trained in LAFP. Therefore, we need training to skillfully deliver the pre-service training*”. There was high turnover of staff. Those who get experienced would leave the institutions frequently.

## Discussion

Overall, most married women in had information about contraceptives, which indicated that the government’s family planning education interventions were successful in raising contraceptive awareness. However, the information was shallow as their knowledge about contraceptive was limited and not consistently translated into behavioral change that leads them to utilize contraceptives as such. This marks that knowledge, but not awareness, is the prime catalyst to contraceptives uptake. It also indicated that universal contraceptive information in the community, in the absence of adequate contraceptive knowledge, did not guarantee effective utilization. The finding regarding contraceptive knowledge gap concurs with related studies conducted elsewhere in Ethiopia [19, 20]. Findings of this study indicated that religion, irrespective of the type, was a major barrier to contraceptive uptake. Although religion has a major influence on a variety of social attitudes, the relationship between religion and its insight on contraceptives remained largely unexplored [21].

According to the national family planning service strategies of FMoH, family planning services are intended to be delivered through community-based, facility-based, social marketing, and outreach approaches. The current study showed that mothers accessed structured contraceptive services primarily at health facilities. In contrast, community-based contraceptive services were fragile which was mainly due to weak home-based contraceptive services which required regular house-to-house visits to each mother. Findings of this study confirmed that only 23% of mothers received house-to-house contraceptive visits by health extension workers in the last one year. A comparable result (20%) was reported in another previous study conducted in Ethiopia based on analysis of the 2000, 2005, and 2011 Demographic and Health Surveys [22].

Findings of this study proved the need of a wider range of investment to improve contraceptive skills of healthcare providers. Findings from key informants and discussants reassured the lack of contraceptive skills among health professionals, particularly health extension workers. Such skills highly lack for LAFP.

## Conclusions

Though the Ethiopian government has so far improved access to contraceptives, utilization is lagging, mainly due to religious influences, limited contraceptives knowledge in the community, and low home-based contraceptive coverage. Societal attitudes and norms of the community towards modern-contraceptives need to be modified through innovative and culturally appropriate interventions. In countries like Ethiopia, where people’s religious devotion remains reasonably high, knowledge on natural-contraceptive methods is equally important to help religious people make an informed decision about family planning in accordance with their faith.

## Additional file

Additional file 1: Data tools in English and local language (Oromiffa). (DOC 516 kb)

## Abbreviations

CSA: Central Statistical Agency
EFMoH: Ethiopian Federal Ministry of Health
EPHA: Ethiopian Public Health Association
IUCD: Intrauterine Contraceptive Devices
LAFP: Long-Acting Family Planning
RH: Reproductive Health
