# Supplementary material for: Socioeconomic and religious differentials in contraceptive uptake in western Ethiopia: a mixed-methods phenomenological study
Source: BMC Womens Health. 2018 Jun 5;18:85. doi: 10.1186/s12905-018-0580-6 (PMC5989360; doi:10.1186/s12905-018-0580-6)
Supplement: Supplementary file 1 — Data tools in English and local language (Oromiffa). (DOC 516 kb) [file 12905_2018_580_MOESM1_ESM.doc]

# Annexes

# Study questionnaire in English

## Annex 1: English Version: Baseline Survey Questionnaire for Assessment of Family Planning Services Utilization

Information Sheet and Consent Form

Good morning/afternoon/evening. My name is _________________________ I am a team member of a research conducted by Ethiopia Public Health Association. We are conducting a study about FP utilization and speaking with women about their family planning service experience and related issues. The results of this survey will be used to mitigate barriers of family planning utilization and improve quality of family planning service delivery.

You have been selected for this interview randomly or by chance, much like picking an orange out of a basket without looking. If you agree to participate you will be asked few questions about yourself, your reproductive and pregnancy history, knowledge, attitude andever and current practice of family planning methods with particular emphasis of LAFP methods. Your participation in this study is completely on voluntary bases means you can refuse to participate in the study entirely or you can refuse to answer any question. There are no anticipated problems you encounter because of your participation in this study. The interview will last approximately 40 -45 minutes.

I would like to inform you that the responses that you provide are very essential not only for the successful accomplishment of the study but also for producing relevant information which will be helpful in improving the delivery of family planning services. The information we collect from you will not be shown to anyone outside of this project.

May I proceed with the questions? Yes --------------- No -----------------

**Interview Information**

Date of interview |__|__| Day |__|__| Month |__|__||__|__| Year

Time started |__|__| Hour |__|__| Minutes

Time ended |__|__| Hour |__|__| Minutes

Result ^*^ |__

Name of interviewer: --------------------------------Sign -------------

Name of the supervisor: --------------------- Date ……../……/…….. Sign ---------------

Checked by ____________________________________

Entered by 1) ____________________________________

2) ____________________________________

*Result Codes:

1=Completed 4=Refused 7= other (specify) ______

2=Not available 5=partly completed

3=Postponed 6=Incapacitated

**Respondent’s Identification**

|  | District | Kebele | Village | HH head | Respondent |
| --- | --- | --- | --- | --- | --- |
| Name |  |  |  |  | ----------- |
| Id (Code) |  |  |  |  |  |

| **Questionnaire Code Number** | **___ /_______/_______** |
| --- | --- |

Section 1: Background characteristics of respondents

| **Q. No** | **Questions** | **Response and their Code** | **Skip** |
| --- | --- | --- | --- |
| 101 | The residence of the interviewee? | 1. ………........Urban 2. …………….Rural |  |
| 102 | How old are you?  (Please tell me your age in years) | ------------­­completed years |  |
| 103 | What is your educational level? | 1. ….No school /unable to read & write 2. …..No school but read and write 3. …..Primary school [grade1-8] 4. …..Secondary school [grade 9-10] 5. …..Preparatory school [grade 11- 12] 6. ….. Diploma 7. …..Degree and above |  |
| 104 | What is the educational level of your partner/husband? | 1.….No school /unable to read & write  2.…..No school but read and write  3…...Primary school [grade1-8]  4.…..Secondary school [grade 9-10]  5.…..Preparatory school [grade 11- 12]  6.…..Diploma  7…..Degree and above  Don’t know …………………………...99 |  |
| 105 | What is your religion? | 1. …………….Orthodox 2. …………….Muslim 3. …………….Catholic 4. …………….Protestant   Others(specify) ………………….88 |  |
| 106 | What is your ethnicity?  Select the major ethnic group | 1. ……………. Oromo 2. ……………. Amhara 3. ……………. Tigre 4. …………………Gurage   Others (specify)…………88 |  |
| 107 | What is your occupation? | 1. ………….Farmer 2. ………….Business Woman (merchant) 3. ………….Government employee 4. …………..Private employee 5. …………...House wife 6. …………….Daily laborer   Others (specify)……………………..88 |  |
| 108 | What is your partner's/husband's occupation? | 1. …………….Farmer 2. …………….Business man(merchant) 3. …………….Government employee 4. …………….Private employee 5. …………….Daily laborer   Others (specify)________________88 |  |
| 109 | How many family members are living in this household? | Family members in number _________ |  |
| 110 | Would you tell me the number of under five children in your family? | Number_____________  Refused to answer………………….99 |  |

Section 2:- Reproductive and Pregnancy History

| Now I would like to ask about all the pregnancies and births you have had during your life | | | |
| --- | --- | --- | --- |
| **Q. no** | **Question and filters** | **Response Code** | **Skip** |
| 201 | Have you ever been pregnant in your life? | 1. ……………….….No 2. …………………..Yes | 1 to 301 |
| 202 | If yes to Q201, how many pregnancies?(including pregnancies that result in abortion) | No of pregnancies__________ |  |
| 203 | Have you ever given birth? (births occurred after 7 months of pregnancy) | 1. …………………….No 2. …………………….Yes | 1 to 207 |
| 204 | If yes to Q 203, how many births? | No of births____________ |  |
| 205 | If yes to Q203, how many of your pregnancies resulted in a baby that was born alive? | No of alive births_________ |  |
| 206 | If yes to Q203, how many of your pregnancies resulted in a baby that was born dead? (still birth) | No of still births___________ |  |
| 207 | Have you ever faced abortion?(pregnancy terminated before 7 months of gestational age) | 1. …………….No 2. …………….Yes | 1 to 301 |
| 208 | If yes to Q207, at what month of the pregnancy? (refer to the recent abortion, if more than one abortion) | |__|__| month of the pregnancy |  |

Section 3: Fertility Preferences

| **Q. no** | | **Questions and Filters** | **Respondents Code** | | **Skip** | |
| --- | --- | --- | --- | --- | --- | --- |
| Now I would like to ask you questions about your family size and expectations. | | | | | | |
| 301 | How many live children do you have? | | | No of live children ___________ | |  |
| 302 | How many children do you think a family should have? | | | |__|__| number   1. As many as the family wants 2. As many as God wants   Don’t know …………………………...99 | |  |
| 303 | Would you like to have more children (than you have now)? | | | 1. …………………………………….No 2. …………………………………….Yes   Don’t know.....………………………….99 | | 1 to 307 |
| 304 | If yes to Q303, how many more children would you like to have? | | | Number ____________  Don’t know ……………………………..99 | |  |
| 305 | If yes to Q303, when you plan to have another birth/child? | | | 1……………………at this time/currently  2…………………After ___/___ years/ months  Don’t know.....….……………………….99 | |  |
| 306 | If yes to Q303 and if you do not plan to become pregnant at this time/currently, what is your main reason? | | | 1. ………………...Lack of money 2. …………………Health concerns 3. ………………….Too much extra work 4. ……………..Fear of pregnancy or delivery 5. ……………….Recently gave birth 6. ……………..No one to take care of child   Other(Please specify)……………………88  Don’t know…............................................99 | |  |
| 307 | If you were to find out today that you were pregnant, how would you feel on a scale from 1 to 5 - one means that you would be very unhappy to have a child now and a five means that you would be very happy to have a child now? | | | 1………………………Very unhappy  2……………………….Unhappy  3……………………….Ambivalent  4……………………….Happy  5……………………….Very happy | |  |
| 308 | Does your husband/partner want the same number of children that you want, or does he want more or fewer than you want? | | | 1. …………………………….. Same number 2. ……………………………... More children 3. ……………………………… Fewer children   Other (Specify) …………………………..88  Don’t know ………………………….99 | |  |

**Section 4: Knowledge, and utilization of family planning methods**

| **Q.no** | **Questions and Filters** | **Response Code** | | | | | | | | | **Skip** | | |
| --- | --- | --- | --- | --- | --- | --- | --- | --- | --- | --- | --- | --- | --- |
| Now I would like to talk about family planning - the various ways or methods that a couple can use to delay or avoid a pregnancy | | | | | | | | | | | | | |
| 401 | Have you ever heard of any family planning method/s? | 1. …………………….No 2. …………………….Yes | | | | | | | | | | | 1 to 405 |
| 402 | If yes, from which source have you heard of the method/s?  (Multiple answers possible) | **Source of information** | | **Yes** | | | | | **No** | | | |  |
|  |  | 1. HEWs | | 1 | | | | | 2 | | | |  |
|  |  | 1. Health professionals | | 1 | | | | | 2 | | | |  |
|  |  | 1. Radio | | 1 | | | | | 2 | | | |  |
|  |  | 1. TV | | 1 | | | | | 2 | | | |  |
|  |  | 1. Neighbor/ Friends   / colleague | | 1 | | | | | 2 | | | |  |
|  |  | 1. Leaflets/broachers/magazines/newspapers | | 1 | | | | | 2 | | | |  |
|  |  | 1. Other (Specify) ---------------------------- | | | | | | | | | | |  |
| 403 | Do you know a place where family planning service is provided? | 1. ……………………….No  2..…………………….….Yes | | | | | | | | | | |  |
| 404 | If yes to Q403, from where can anyone obtain a method of family planning? | 1.………….…….Health Post  2.……………..…Health Center  3.…………….….District hospital  4.…………..…….Zonal hospital  5.………………...Private hospital  6.……………..….Private clinic  7.………………….Private drug store  8.……………….….Pharmacy  Other (specify) ………………...…..88 | | | | | | | | | | |  |
| 405 | Which method of family planning have you ever heard? (First let the respondent describe and then ask the respondent by reading the choices)  (Multiple answers possible) |  | | | | Yes | | | | | | No |  |
|  |  | Female Sterilization/Tubal ligation | | | | 1 | | | | | | 2 |  |
|  |  | Male sterilization/Vasectomy | | | | 1 | | | | | | 2 |  |
|  |  | Pills(COC, POP) | | | | 1 | | | | | | 2 |  |
|  |  | Injectable /Depo-Provera | | | | 1 | | | | | | 2 |  |
|  |  | IUCD | | | | 1 | | | | | | 2 |  |
|  |  | Implants | | | | 1 | | | | | | 2 |  |
|  |  | Condoms | | | | 1 | | | | | | 2 |  |
|  |  | Diaphragm/Foam/Jelly | | | | 1 | | | | | | 2 |  |
|  |  | Standard days method | | | | 1 | | | | | | 2 |  |
|  |  | Lactation amenorrhea method | | | | 1 | | | | | | 2 |  |
|  |  | Rhythm/ Calendar method | | | | 1 | | | | | | 2 |  |
|  |  | Withdrawal method | | | | 1 | | | | | | 2 |  |
|  |  | Abstinence | | | | 1 | | | | | | 2 |  |
|  |  | Emergency contraception | | | | 1 | | | | | | 2 |  |
| 406 | Have you ever used any family planning (FP) method or tried in any way to delay or avoid getting pregnant? | 1. ……………………….No 2. ……………………….Yes | | | | | | | | | | | 1 to 409 |
| 407 | If yes to Q406, at what age did you first start using any method of contraception? | Age |__|__|  Don’t remember/know …….99 | | | | | | | | | | |  |
| 408 | If yes to Q406, what was/were the first method/s you used?  (Multiple answers possible) | 1. … 1……………………..…Pills 2. 2. 2…………………….… Injectables   …….. 3. ………………………Implants  4…………………….…Condom   1. … 5………………….........IUCD 2. … 6………………….…….Female sterilization   …….. 7. ………………………Male sterilization  8………………………...Diaphragm/foam/jelly   1. ……..9………………………….Rhythm method 2. … 10…………………………Withdrawal 3. …… 11…………………..Exclusive breastfeeding/LAM 4. Other Others (specify)___________________88 | | | | | | | | | | |  |
| 409 | Are you (or your spouse/partner) currently using any method to delay or avoid getting pregnant? | 1. ……………………………………...No 2. ……………………………………...Yes   Don’t know ………………………….. 99 | | | | | | | | | | | **1 to 421** |
| 410 | If yes to Q409, which contraceptive method you are currently using to delay or avoid getting pregnant? |  | | 1. Yes | | | | | | 1. No | | |  |
|  |  | 1………..….Female sterilization | | 1 | | | | | | 2 | | |  |
|  |  | 1. ……………Pills | | 1 | | | | | | 2 | | |  |
|  |  | 1. …………….Injectable | | 1 | | | | | | 2 | | |  |
|  |  | 1. ………………Implants | | 1 | | | | | | 2 | | |  |
|  |  | 1. ………………IUCD | | 1 | | | | | | 2 | | |  |
|  |  | 1. …………….Condom | | 1 | | | | | | 2 | | |  |
|  |  | 1. ….Diaphragm/foam/jelly | | 1 | | | | | | 2 | | |  |
|  |  | 1. ……….Rhythm method | | 1 | | | | | | 2 | | |  |
|  |  | 1. ……….Withdrawal | | 1 | | | | | | 2 | | |  |
|  |  | 1. …….Exclusive breastfeeding | | 1 | | | | | | 2 | | |  |
|  |  | Other (Please specify___________88 | | | | | | | | | | |  |
| 411 | For how many years/months you have been using the current contraceptive method (Name the method from Q408) without stopping? | Month . . ................................................. |__|__|  Year……..………………………… |__|__||__|__| | | | | | | | | | | |  |
| 412 | For what purpose you are using the methods? | 1. …………………..…………… Limiting birth 2. ……………………………..…Spacing birth   Others (specify) …………………………..88 | | | | | | | | | | |  |
| 413 | From where (which place) you have gotten the current contraceptive method you are using? | 1. ………………………Health post /HEW 2. ……………………...Governmet Health Centre 3. ………………….…..Government Hospital 4. ………………………Private clinic 5. ……………………….Private Hospital 6. ……………………….Private drug store 7. …………………….…..Pharmacy 8. ……………………..…NGO health facilities   Others(specify)___________________88 | | | | | | | | | | |  |
| 414 | Why do you choose that facility? | 1. …………..Close to my home 2. …………..Employer designated site 3. …………..Affordable 4. …………..Doctor is available 5. …………..Provider seems knowledgeable 6. …………..Provider is friendly 7. …………..Clean facilities 8. …………..Adequate medications in stock 9. …………..Needed service only available here 10. …………..Good reputation 11. …………..Short waiting time 12. …………..I know the staff 13. ………….Always come here 14. ………….Friends or relatives recommend 15. ………...Services are given with sufficient privacy 16. ………...No particular reason   Other (specify….........................…..88  Don’t know…………………………..99 | | | | | | | | | | |  |
| 415 | At that time (when you have taken the method you are using now) have you been counseled about FP/ contraceptive methods? | 1. …………..……………….….….No 2. ………………..…………….…..Yes   Do not remember……………..….….77 | | | | | | | | | | |  |
| 416 | If yes to Q415, on which topic/issue you have you been informed by the counselor about the method you chosen and currently using?  (Multiple answers possible) | 1. Effectiveness 2. Advantage and disadvantages 3. Side effects or problems related to the method and what to do if experience side effects 4. How to use the method/instructions 5. When to return   Other (specify….........................….88  Do not remember…………….…….77 | | | | | | | | | | |  |
| 417 | How effective do you think the method you are currently using in preventing pregnancy? | 1. ……………………………Very effective 2. ……………………………Effective 3. ……………………………Some what effective 4. ……………………………Not effective at all   Donot know ………………….99 | | | | | | | | | | |  |
| 418 | How did you get the method you are using? | 1. ………..……...free of payment 2. ………………..with payment | | | | | | | | | | |  |
| 419 | Do you/your partner experience any difficulty in getting/the method you are currently using? | 1. …………………………..No 2. …………………………..Yes   Do not remember……………..77 | | | | | | | | | | | **1, 77 to 421** |
| 420 | What difficulties did you experience?  (Multiple answers are possible). | 1. ….the facility where I got the service is far away   or inconvenient to access   1. …Is expensive to purchase 2. …Had difficulty of being seen/served by facility workers 3. …Encounter irregular or out-of-stock contraceptive supplies 4. …Experienced family or social resistance 5. …Religious pressure 6. …There is transportation problem 7. …There is time constraint   Other (Please specify)__________________88 | | | | | | | | | | |  |
| 421 | From which facility do you prefer to get the contraceptive method you are currently using?  (Multiple answers possible) | 1. …………………….…...Health Post 2. ……………………….…Health Center 3. ………………………….District hospital 4. …………………………..Zonal hospital 5. ………………………..…Private clinic 6. ……………………….….Private drug store 7. ………………………...…Pharmacy 8. ……………………………Private hospital   Other (specify) ………..……..88 | | | | | | | | | | |  |
| 422 | Is there another contraceptive method that you would prefer to use? | 1. ……………………………..No 2. …………………………....Yes   Don’t know………………..…...99 | | | | | | | | | | | 1,99to 424 |
| 423 | If yes to Q422, what would this method be? | 1. …………………….Female sterilization 2. …………………….Male sterilization 3. …………………....Pill 4. …………………….IUCD 5. ……………………Implants 6. …………………….Injectables 7. ……………………Condom 8. ……………………Diaphragm/foam/jelly 9. ……………………..Rhythm method 10. .Withdrawal 11. ………………………….Prolonged breastfeeding   Other(Please specify)__________________88 | | | | | | | | | | |  |
| 424 | In the last 12 months, were you visited by a HEW/VCHW or others who talked to you about family planning? | 1. ……………………………..…….No 2. ……………………………………Yes   Do not remember ……………………77 | | | | | | | | | | |  |
| 425 | In the last 12 months, have you visited a health facility for family planning services? | 1. ………………………..….No 2. …………………………….Yes | | | | | | | | | | |  |
| 426 | Who made decision to seek contraceptive methods? | 1. ……………………Mainly respondent 2. ……………………Mainly husband/partner 3. ……………………Joint decision 4. ……………………Some other person   Other (Please specify)___________________88 | | | | | | | | | | |  |
| 427 | Are there women whom you know who are not using any method of contraception? | 1. ……………….No 2. ……………….Yes   Do not know ………………..99 | | | | | | | | | | | 2, 99 to 429 |
| 428 | If yes, what is/are your perception/ their reasons if you heard from them/ for not using any family planning method? | 1. …… Facility that provide the service is far away 2. …….Transportation problem or inconvenient to   Access   1. ………Want more children 2. ……….Is/are unable to purchase 3. …………Fear of side effects/complications 4. …………unwelcoming approach of service   providers/poor quality of care   1. …………Long waiting time 2. …………Lack knowledge about other contraceptive options 3. ……………Sexual partner resistance 4. ………...Experienced family or social resistance 5. ………..…Religious influence 6. ………….There is transportation problem 7. ……….…There is time constraint   Other (Please specify)_________________88 | | | | | | | | | | |  |
| 429 | Have you ever heard about LAFP methods Implants/ implanon? | Implants/implanon | 1. No | | | | | 1. Yes | | | | | 1 to both of them  > 450 |
|  |  | Intrauterine contraceptive device(IUCD) | 1. No | | | | | 1. Yes | | | | |  |
| 430 | If yes to Q429,for Implants, IUCD or both, from whom have you heard of it (what is your source of information)  Multiple response is possible | 1. ………………………HEWs/CHWs 2. …………………….…Health Professionals 3. ………………………...Sexual partner/husband 4. ……………………..…Radio 5. ………………………..TV 6. ………………………Printed materials-leaflet,   magazine, newspaper   1. ………………………Family members 2. ……………………….Neighbor 3. ………………………...Friend   Other (Specify) _____________________88 | | | | | | | | | | |  |
| 431 | Have you ever used implant (Implanon)? | 1. ………………………..…No 2. ……………………….….Yes | | | | | | | | | | | **1 to 437** |
| 432 | If yes to Q 430, by whom it was inserted? | 1. ………………………. By HEWs 2. …………………….…By Health Professionals | | | | | | | | | | |  |
| 433 | For how long you have been using the implanon? | Month . . . . . ................................... |__|__|  Year . . …………………………… |__|__||__|__|  Do not know …………………………….…99 | | | | | | | | | | |  |
| 434 | Where (which facility) it was inserted? | 1. …………………..….Health Post 2. …………………..... Health Center 3. …………………….. Government hospital 4. ………………………Private clinic 5. …………………….... Private hospital 6. ………………………..NGO health facility   Other (specify) ………………...…..88  Don’t know………………………...99 | | | | | | | | | | |  |
| 435 | Are you still using implant (implanon)? | 1. …………………………….……No 2. ……………………………….…Yes | | | | | | | | | | | **2 to 438** |
| 436 | If no, why is it taken out? | 1. ………………Want more children? 2. …………….…Partner/ husband opposition 3. ………………..Pressure from family members 4. ………………..Religious influence? 5. ………….….Fear of side effects/complications 6. ………. ……..Due to rumors I heard from other   people   1. ……………….The methods fail? 2. ………………. End of prevention period   Others (Specify) _________________88 | | | | | | | | | | |  |
| 437 | If you are not using implanon what are the reasons? | 1…..…….…Facility that provide the service is far  2……..……..Transportation problem or inconvenient  to access   1. ………….Is expensive to purchase 2. …………. Unwelcoming approach from service   providers/ long waiting time  5…………….Encounter irregular or out-of-stock  contraceptive supplies  6……… ……Lack awareness about it  7……………..Fear of side effects  8……………. Experienced family or social resistance   1. …………....Religious views   10……….……There is time constraint  11.……………. Due to rumors I heard from other  people   1. Other (Please specify)_______________88 | | | | | | | | | | |  |
| 438 | Concerning Implants/ Implanon, which information/knowledge do you have? | Norplant can prevent pregnancy for 5 years | | | | | 1. No 2. Yes | | | | | |  |
|  |  | Implanon(single rod) can prevent pregnancy for 3 years | | | | | 1. No 2. Yes | | | | | |  |
|  |  | Implants require minor surgical procedure during insertion and removal | | | | | 1. No 2. Yes | | | | | |  |
|  |  | Implants is immediately reversible(becomes pregnant quickly when removed) | | | | | 1. No 2. Yes | | | | | |  |
| 439 | Do you know the facility where Implanonis readily available and provided? | 1. ……………………………….……No 2. ……………………………………Yes | | | | | | | | | | |  |
| 440 | If yes, where a woman who wants to use Implanon can get the service? | 1. …………………Health post by HEWs 2. ………………….Health centre 3. …………………..Private clinic 4. …………………..Government Hospital 5. ……………………Private Hospital   Other (Please specify)________________88 | | | | | | | | | | |  |
| 441 | Have you ever used IUCD? | 1…………………..………………No  2………………….………………Yes | | | | | | | | | | |  |
| 442 | If yes, by whom it was inserted? | 1. …………………. By HEWs 2. ………………..…By Health Professionals | | | | | | | | | | |  |
| 443 | For how long have you been using the IUCD? | Month . . . . . ....................................... |__|__|  Year……………………………… |__|__||__|__|  Do not know ……………………….…99 | | | | | | | | | | |  |
| 444 | From where (which facility) was it inserted? | 1.…………………..….Health Post  2.…………………..... Health Center  3.…………………….. Government hospital  4………………………Private clinic  5…………………….... Private hospital  6………………………..NGO health facility  Other (specify) ……………....88  Don’t know…………………..99 | | | | | | | | | | |  |
| 445 | Are you still using IUCD? | 1. …………………………….……No 2. ……………………………….…Yes | | | | | | | | | | |  |
| 446 | If no, why is it taken out? | 1. ………………Want more children? 2. …………….…Partner/ husband opposition 3. ………………..Pressure from family members 4. ………………..Religious influence? 5. ………….….Fear of side effects/complications 6. ………. ……..Due to rumors I heard from other   people   1. ……………….The methods fail? 2. ………………. End of prevention period   Others (Specify) _________________88 | | | | | | | | | | |  |
| 447 | If you are not using IUCD what are the reasons | 1…..…….…Facility that provide the service is far  2……..……..Transportation problem or inconvenient  to access   1. ………….Is expensive to purchase 2. …………. Unwelcoming approach from service   providers/ long waiting time  5…………….Encounter irregular or out-of-stock  contraceptive supplies  6……… ……Lack awareness about it  7……………..Fear of side effects  8……………. Experienced family or social resistance   1. …………....Religious views   10……….…..There is time constraint  11.…………. Due to rumors I heard from other  people   1. Other (Please specify)_______________88 | | | | | | | | | | |  |
| 448 | Concerning IUCD, which information/knowledge do you have? | IUCD can prevent pregnancies for more than 10 years | | | 1.. No  2. Yes | | | | | | | |  |
|  |  | IUCD is not appropriate for female at high risk of getting STIs | | | 1. No 2. Yes | | | | | | | |  |
|  |  | IUCD has no interference with sexual intercourse or desire | | | 1. No 2. Yes | | | | | | | |  |
|  |  | IUCD is immediately reversible(become pregnant quickly when removed) | | | 1. No 2. Yes | | | | | | | |  |
|  |  | IUCD cannot cause cancer | | | 1. No 2. Yes | | | | | | | |  |
| 449 | Do you know the facility where IUCD is readily available and provided? | 1. ……………………………….……No 2. ……………………………………Yes | | | | | | | | | | |  |
| 450 | If yes, where a woman who wants to use Implanon can get the service? | 1. ………………….Health post by HEWs 2. ………………….Health centre 3. …………………..Private clinic 4. …………………..Government Hospital 5. ……………………Private Hospital   Other (Please specify)________________88 | | | | | | | | | | |  |
| 451 | Do you have a plan to use contraceptives in the future? | 1. …………………………………..No 2. …………………………………..Yes | | | | | | | | | | |  |
| 452 | If yes, for what purpose? | 1. ………….Spacing 2. …………..limiting   Other (Please specify)_______________88 | | | | | | | | | | |  |
| 453 | Have you discussed about family planning with anybody (out of health professionals) in the last 12 months? | 1. ……………………………………No 2. ……………………………………Yes | | | | | | | | | | |  |
| 454 | If yes, with whom you usually discussed? | 1. ………………….....Husband/ partner 2. ………………….…Family members 3. …………………….Friends 4. …………………….Neighbors   Other (please specify) ___________________88 | | | | | | | | | | |  |
| 455 | Who in your family usually has the final say onobtaining own healthcare? | 1. Respondent alone 2. Respondent and husband jointly 3. Respondent and someone else 4. Husband/ partner 5. Someone else in the household. | | | | | | | | | | |  |

## Study questionnaire in local language: Oromiffa

## Waldaa Fayyaa Haawaasaa Itiyoophiyaa

Gaaffilee bu’ura qo’annoo ittifayyadama tajaajila karoora maatii

**Guuca Odeeffannoo fi eeyyama**

Akkam bultan/oltan. Maqaan koo _____________________. Ani miseensa garee qo’annoo Waldaa Fayyaa Haawaasaa Itiyoophiyaan gaggeefamu dha. Nuti waa’ee qo’annoo ittifayyadama karoora maatii fi dubartoota waliin immoo waa’ee muxxannoo tajaajila karoora maatii isaanii fi dhimmoota kanaan walqabatan irratti dubbanna. Bu’aan qo’annoo kanaa danqaalee ittifayyadama karoora maatii hir’dhisuu fi qulqullina keenninsa tajaajila karoora maatii foyyeesuufi.

Atii gaafatamuuf kan filatamtee akka carraatii, akka zanbiila keessaa osoo hin ilaaliin buurtukaana tokko kaasuutti. Hirmaachuuf yoo eeyyama kee ta’e gaaffilee xiqqoo waa’ee keetii, seenaa walhormaataa fi ulfa kee, beekumsa, ilaalcha fi ittifayyadama gosoota karoora maatii kanaan dura fi yeroo ammaa keesumaahu gosoota karoora maatii yeroo dheeraa irratti xiyyeefachuudhaan gaafatamta. Qo’annoo kana irratti hirmaannaan kee gutumaa gutuutti feedhii irratti kan hundaa’ee dha, jachuun akka jiruutti qo’annoo kana irratti hirmaachuu dhisuu dandeessa yokiin gaafii barbaadde deebisuu dhisuu dandeessa. Qo’annoo kana irratti hirmaachuu keetiin rakkon kamiyyuu sirra hingahu. Gaaffileen kuun tilmaamaan daqiqaa 40-45 fudhatan.

Deebiin atii naaf keennituu baayyee barbaachisaa ta’u isaa sitti himuu barbaada kunis milkaa’ina xumura qo’annoo kanaaf qofa osoo hinta’in odeeffannoo kanaan walqabatan maddisisuu fi keenninsa tajaajila karoora maatii foyyeesuuf gargaara. Odeefannon sirraa fudhanne piroojaktii kanaan ala nama kamiyyufuu hinagarsifamu.

Gaaffilee itti fuufuu nandanda’aa? Eeyyee__________ Lakki__________

Galatoomi

**Odeefannoo gaaffi**

Guyyaa gaaffi Guyyaa /___/___/ Ji’a /___/___/ Bara /___/___/___/___/

Yeroo ittijalqabame Sa’aa /___/___/ Daqiqaa /___/___/

Yeroo ittixumurame Sa’aa /___/___/ Daqiqaa /___/___/

Bu’aa* /___

Maqaa gaafataa:________________________________ Mallattoo________________

Maqaa Suparvaayzara___________________________ Guyyaa ___/___/___ Mallattoo______

Kan ilaale ________________________________________

Kan galchee 1,______________________________________

2,______________________________________

*Koodiilee Bu’aa

1= Guutuudha 4= Deebisuu hinbarbaanne 7= Kan biro (ibsi)_______

2= Hinargamne 5= Hammi ta’e guutuudha

3= Yeroo biraatiif darbeera 6= Hindandeenye

Eenymaa Gaafatamtu

|  | Aanaa | Ganda/Araddaa | Garee | Abbaa/Haadha warraa | Gaafatamtuu |
| --- | --- | --- | --- | --- | --- |
| Maqaa |  |  |  |  | _________ |
| Koodii |  |  |  |  |  |

| **Lakkofsa koodii gaaffi** | ______/_______/_________ |
| --- | --- |

**Kuutaa 1: Odeeffannoo gaafatamtootaa**

| **Lakk Gaafi** | **Gaaffilee** | **Deebii fi koodii isaani** | **Darbi** |
| --- | --- | --- | --- |
| 101 | Iddoo jireenya gaafatamtu? | 1.-----------Magaalaa  2.-----------Baadiyyaa |  |
| 102 | Umriin kee meeqa? (Umrii kee waggaan natti himi) | Waggaa ------------guuteera |  |
| 103 | Sadarkaan barumsa keetii meeqa? | 1.----Hinbaranne/dubbisuu fi barressu hindanda’u  2.----Hinbaranne garu nan dubbisa, nan barressa  3.----Sadarkaa 1ffaa[Kutaa 1-8]  4.----Sadarkaa 2ffaa [Kutaa 9-10]  5.----Sadarkaa Qophaa’inaa [Kutaa 11-12]  6.----Dipiloomaa  7.----Digrii fi isaa ol |  |
| 104 | Sadarkaan barumsa abbaa warraa keetii meeqa? | 1.----Hinbaranne/dubbisuu fi barressu hindanda’u  2.----Hinbaranne garu nan dubbisa, nan barressa  3.----Sadarkaa 1ffaa[Kutaa 1-8]  4.----Sadarkaa 2ffaa [Kutaa 9-10]  5.----Sadarkaa Qophaa’inaa [Kutaa 11-12]  6.----Dipiloomaa  7.----Digrii fi isaa ol  Hinbeeku-----------------------------------------99 |  |
| 105 | Amantaan kee maali? | 1.----------Ortoodooksii  2.----------Muusliima  3.----------Kaatoolika  4.----------Prooteestaantii  Kan biroo(ibsi)---------------------------------88 |  |
| 106 | Sabnii kee maali? | 1.----------Oromoo  2.----------Amaara  3.----------Tigree  4.----------Guraagee  Kan biroo(ibsi)----------------------------------88 |  |
| 107 | Hojiin kee maali? | 1.----------Qonnaan Bultu  2.----------Daldaltu  3.----------Hojjettu mootummaa  4.----------Hojjettu dhunfaa  5.----------Haadha manaa  6.----------Hojjettu guyyaa  Kan biroo(ibsi)---------------------------------88 |  |
| 108 | Hojiin abbaa warraa keetii maalii? | 1.----------Qonnaan Bulaa  2.----------Daldalaa  3.----------Hojjetaa mootummaa  4.----------Hojjetaa dhunfaa  5.----------Hojjetaa guyyaa  Kan biroo(ibsi)---------------------------------88 |  |
| 109 | Mana kana keessa miseensoota maatii meeqaatu jira? | Lakkofsa miseensoota maatii____________ |  |
| 110 | Lakkofsa ijoolee waggaa shan gadi maatii keessan keessa jiran natti himtaa? | Lakkofsa____________________  Deebisuu hinbarbaanne----------------------99 |  |

Kutaa 2: Seenaa walhormaataa fi ulfaa

| Amma immo waa’ee ulfaa fi da’umsa umrii kee keessatti siqunname hunda sigaafa dha | | | |
| --- | --- | --- | --- |
| **Lakk Gaafi** | **Gaaffilee fi calallii** | **Koodii Deebii** | **Darbi** |
| 201 | Umrii kee keessatti ulfa taatee beektaa? | 1.-----------Lakki  2.-----------Eeyyee | 1 gara 301 |
| 202 | Gaaffin 201 eeyyee yoo ta’e ulfa meeqa? (ulfa bahee dabalatee) | Lakkofsa ulfaa___________ |  |
| 203 | Kanaan dura deessee beektaa? (da’umsa turmaata ulfa ji’a 7 boodde ta’e) | 1.-----------Lakki  2.-----------Eeyyee | 1 gara 207 |
| 204 | Gaaffin 203 eeyyee yoo ta’e, yeroo meeqa deessee? | Lakkofsa da’umsaa_________ |  |
| 205 | Gaaffin 203 eeyyee yoo ta’e, ulfa meeqa irraa ijoolleen lubbu qaban dhalatan? | Lakkofsa Ijoolee lubbu qaban dhalatan_________ |  |
| 206 | Gaaffin 203 eeyyee yoo ta’e, ulfa meeqa irraa ijoolleen lubbu hinqabne/du’an dhalatan? | Lakkofsa Ijoolee lubbu hinqabne/du’an dhalatan_________ |  |
| 207 | Kanaan dura ulfi sirraa bahee beekaa? ( ulfa ji’a 7 osoo hingutin bahe) | 1.-----------Lakki  2.-----------Eeyyee | 1 gara 301 |
| 208 | Gaaffin 207 eeyyee yoo ta’e, ji’a meeqafaatti? (yoo ulfi bahee tokko ol ta’e isa dhumaa fudhadhu) | ji’a ulfa /_____/_____/ |  |

Kutaa 3: Filanno ijoolee godhachu

| Amma immo waa’ee baayyina maatii kee fi maal akka eegdu sigaafa dha | | | |
| --- | --- | --- | --- |
| **Lakk Gaafi** | **Gaaffilee fi calallii** | **Koodii Deebii** | **Darbi** |
| 301 | Ijoollee lubbun jiran meeqa qabda? | Lakkofsa ijoolle lubbun jiran__________ |  |
| 302 | Maatiin tokko ijoollee meeqa qabaachu qaba jatte yaadda? | Lakkofsa /____/____/  1.Baayyina akka maatiin barbaadeetti  2.Baayyina akka waaqni barbaadeetti  Hinbeeku--------------------------------------99 |  |
| 303 | Ijoollee amma qabduun caala dabalataan ijoollee godhachuu barbaaddaa? | 1.-----------Lakki  2.-----------Eeyyee  Hinbeeku------------------------------------------99 | 1 gara 307 |
| 304 | Gaaffin 303 eeyyee yoo ta’e, ijoollee meeqa dabalachuu barbaadda? | Lakkofsa________________  Hinbeeku------------------------------------------99 |  |
| 305 | Gaaffin 303 eeyyee yoo ta’e, yoom ijoollee godhachuuf karoorfatte? | 1.----------- yeroo kana / amma  2.-----------waggaa/ji’a _____/____ booda  Hinbeeku------------------------------------------99 |  |
| 306 | Gaaffin 303 eeyyee yoo ta’e fi yeroo kana/amma ulfa ta’u yoo hin karoorfanne sababni kee maali? | 1.--------- Qarshii hinqabu  2.--------- Fayyaan walqabatee wantootni yaaddeesan waan jiranif  3.----------Hojii dabalataa baayyee waan qabuf  4.---------- Ulfa fi da’umsa waan sodaadhuf  5.---------- Yeroo dhihootti waanan daheef  6.---------- Namni daa’ima kunuunsu waan hinjireef  Kan biroo(ibsi)------------------------------------88  Hinbeeku------------------------------------------99 |  |
| 307 | Silaa har’a ulfa taatee turtee, maaltuu sitti dhagahaama qabxii 1 hanga 5 keenni – tokko jachuun yeroo ammaa daa’ima godhachuu keetiin baayyee gadita fi shan jachuun immo yeroo ammaa daa’ima godhachuu keetiin baayyee gammadda | 1.--------- Baayyeen gadda  2.--------- Nan gadda  3.---------Walmakaa dha(gaddu fi gammadu)  4.---------- Nan gammada  5.---------- Baayyeen gammada |  |
| 308 | Abbaan warraa kee lakkofsuma ijoollee ati barbaaddhu barbaada, moo kan ati barbaaddu caala yokin kan ati barbaaddu gadi barbaada? | 1.--------- kan ani barbaadu barbaada  2.--------- Nacaala barbaada  3.---------- kan ani barbaadu gadi  Kan biroo (ibsi)---------------------------------88  Hinbeeku ----------------------------------------99 |  |

Kutaa 4: Beekumsaa fi ittifayyadama gosa/mala karoora maatii

| Amma immo waa’ee karoora maatii – maloota adda addaa namoonni walinjiraatan ulfa tursisuuf yokin ulfa ittisuuf fayyadaman walindubbanna. | | | | | | | | | | | | |  |
| --- | --- | --- | --- | --- | --- | --- | --- | --- | --- | --- | --- | --- | --- |
| **Lakk Gaafi** | **Gaaffilee fi calallii** | **Koodii Deebii** | | | | | | | | | | **Darbi** |  |
| 401 | Kanaan dura waa’ee gosa karoora maatii kamiyyu dhageessee beektaa? | 1.-----------Lakki  2.-----------Eeyyee | | | | | | | | | | 1 gara 405 |  |
| 402 | Gaaffin 401 eeyyee yoo ta’e, eessa irraa dhageessee?  (Deebiin tokko ol ta’u danda’a) | **Madda odeeffannoo** | | | | Eeyyee | | | | | Lakki |  |  |
|  |  | 1.HEF(Hojjet.Ekist.Fayyaa) | | | | 1 | | | | | 2 |  |  |
|  |  | 2.Ogeesoota fayyaa | | | | 1 | | | | | 2 |  |  |
|  |  | 3.Raadiyoo | | | | 1 | | | | | 2 |  |  |
|  |  | 4.TV | | | | 1 | | | | | 2 |  |  |
|  |  | 5.Ollaa/Hiriyoota/Michuu | | | | 1 | | | | | 2 |  |  |
|  |  | 6.Barrefamoota adda addaa, barulee, gaazeexaa, kitaaba | | | | 1 | | | | | 2 |  |  |
|  |  | 7.Kan biroo (ibsi)----------------------------- | | | | | | | | | |  |  |
| 403 | Iddoo tajaajilli karoora maatii itti keennamu beektaa? | 1.-----------Lakki  2.-----------Eeyyee | | | | | | | | | |  |  |
| 404 | Gaaffin 403 eeyyee yoo ta’e, namni kamiyyu gosa karoora maatii eessaa argachu danda’a? | 1.--------- Keellaa Fayyaa  2.--------- Buufata Fayyaa  3.---------- Hospitaala Aanaa  4.---------- Hospitaala Godinaa  5.---------- Hospitaala dhunfaa  6.---------- Kilinika dhunfaa  7. ---------- Kuusaa qoricha dhunfaa  8.----------- Mana qorichaa  Kan biroo(ibsi)------------------------------------88 | | | | | | | | | |  |  |
| 405 | Kanaan dura waa’ee gosa karoora maati kami dhageessee? (Jalqaba gaafatamtuun akka tarreesitu godhi sanaan booda dubbisuudhaan gaafatamtuun akka filattu godhi) |  | | Eeyyee | | | | | | Lakki | |  |  |
|  |  | Dhaabbi kan dubartootaa (Tubal ligation) | | 1 | | | | | | 2 | |  |  |
|  |  | Dhaabbi kan dhiraa (Vasectomy) | | 1 | | | | | | 2 | |  |  |
|  |  | Kiniina | | 1 | | | | | | 2 | |  |  |
|  |  | Marfee ji’a sadin keennamu | | 1 | | | | | | 2 | |  |  |
|  |  | Gadaameesa keessa kan taa’u (IUCD) | | 1 | | | | | | 2 | |  |  |
|  |  | Hirree harkaa jala kan galu (Implants) | | 1 | | | | | | 2 | |  |  |
|  |  | Koondoomii | | 1 | | | | | | 2 | |  |  |
|  |  | Gadaameesa keesa kan kaawamu(Diaphragm, Foam,Jelly) | | 1 | | | | | | 2 | |  |  |
|  |  | Guyyota murteesson fayyadamu (Standard days mthod) | | 1 | | | | | | 2 | |  |  |
|  |  | Harma hosiisudhaan xuriin akka hindhufnee fyyadamu (LAM) | | 1 | | | | | | 2 | |  |  |
|  |  | Guyyota lakaa’un fayyadamu (Rhythm/Calendar method) | | 1 | | | | | | 2 | |  |  |
|  |  | Sanyii dhiraa nafa dubarti alatti dhangalaasuu (Withdrawal Method) | | 1 | | | | | | 2 | |  |  |
|  |  | Qunnamtii saalaa irra ofqusachuu (Abstinence) | | 1 | | | | | | 2 | |  |  |
|  |  | Karoora maatii tasaa | | 1 | | | | | | 2 | |  |  |
| 406 | Kanaan dura gosa karoora maatii fayyadamtee beektaa yokin ulfa tursisuuf yokin ulfa ittisuuf yaaltee jirtaa? | 1.-----------Lakki  2.-----------Eeyyee | | | | | | | | | | 1 gara 409 |  |
| 407 | Gaaffin 406 eeyyee yoo ta’e, gosa karoora maatii jalqabaa waggaa meeqafaa kee irratti fayyadamtee? | Umrii /____/____/  Hin yaadadhu/hinbeeku-------------------------99 | | | | | | | | | |  |  |
| 408 | Gaaffin 406 eeyyee yoo ta’e, gostii karoora maatii jalqaba fayyadamtee maalii?  (Deebiin tokko ol ta’u danda’a) | 1.----Kiniina  2.----Marfee ji’a sadin keennamu  3.----Hirree harkaa jala kan galu (Implants)  4.----Koondoomii  5.----Gadaameesa keessa kan taa’u (IUCD)  6.----Dhaabbi kan dubartootaa (Tubal ligation)  7.----Dhaabbi kan dhiraa (Vasectomy)  8.----Gadaameesa keesa kan kaawamu (Diaphragm, Foam,Jelly)  9.----Guyyota lakaa’un fayyadamu (Rhythm/Calendar method)  10.----Sanyii dhiraa nafa dubartii alatti dhangalaasuu (Withdrawal Method)  11.----Harma hosiisudhaan xuriin akka hindhufnee fyyadamu (LAM)  Kan biroo(ibsi)----------------------------------88 | | | | | | | | | |  |  |
| 409 | Ati yokin abbaawarraan kee yeroo ammaa ulfa tursisuuf yokin ittisuuf gosa karoora maatii fayyadamaa jirtuu? | 1.-----------Lakki  2.-----------Eeyyee  Hinbeeku------------------------------------------99 | | | | | | | | | | 1 gara 421 |  |
| 410 | Gaaffin 409 eeyyee yoo ta’e, yeroo ammaa ulfa tursisuuf yokin ittisuuf gosa karoora maatii kam fayyadamaa jirta? |  | | | Eeyyee | | | | Lakki | | |  |  |
|  |  | 1.--Dhaabbi kan dubartootaa (Tubal ligation) | | | 1 | | | | 2 | | |  |  |
|  |  | 2.--Kiniina | | | 1 | | | | 2 | | |  |  |
|  |  | 3.--Marfee ji’a sadin keennamu | | | 1 | | | | 2 | | |  |  |
|  |  | 4.--Hirree harkaa jala kan galu (Implants) | | | 1 | | | | 2 | | |  |  |
|  |  | 5.--Gadaameesa keessa kan taa’u (IUCD) | | | 1 | | | | 2 | | |  |  |
|  |  | 6.--Koondoomii | | | 1 | | | | 2 | | |  |  |
|  |  | 7.--Gadaameesa keesa kan kaawamu (Diaphragm, Foam, Jelly) | | | 1 | | | | 2 | | |  |  |
|  |  | 8.--Guyyota lakaa’un fayyadamu (Rhythm/Calendar method) | | | 1 | | | | 2 | | |  |  |
|  |  | 9.--Sanyii dhiraa nafa dubartii alatti dhangalaasuu (Withdrawal Method) | | | 1 | | | | 2 | | |  |  |
|  |  | 10.--Haarma qofa hosiisuu | | | 1 | | | | 2 | | |  |  |
|  |  | Kan birooibsi)---------------------------------88 | | | | | | | | | |  |  |
| 411 | Waggaa/ji’oota meeqaaf gosa karoora maatii (maqaa isaanii gaaffi 408 irraa osoo addaan hinkutin dubbisiif) amma fayyadama jirtu fayyadamtee? | Ji’a----------------------------/____/____/  Waggaa--------------------/____/____//____/____/ | | | | | | | | | |  |  |
| 412 | Gosa karoora maatii kaayyoo maalif fayyadamaa jirta? | 1.------------- Da’umsa dhaabuuf  2.------------- Da’umsa addaan fageessuf  Kan biroo(ibsi)-------------------------------------88 | | | | | | | | | |  |  |
| 413 | Gosa karoora maatii amma fayyadamaa jirtu eessaa argatte? | 1.-------Keellaa Fayyaa/HEF  2.------- Buufata Fayyaa mootummaa  3.------- Hospitaala mootummaa  4.------- Kilinika dhunfaa  5.------- Hospitaala dhunfaa  6.------- Kuusaa qoricha dhunfaa  7.------- Mana qorichaa  8.------- Dhaabbilee fayyaa miti-mootummaa  Kan biroo(ibsi)--------------------------------88 | | | | | | | | | |  |  |
| 414 | Dhaabbata fayyaa sana maaliif filatte? | 1.----- Mana jireenyaa kootitti dhiyaata  2.----- Hojjetootni iddoo qopheesani  3.----- Gatii salphaan argama  4.----- Doktaroonni ni argamu  5.----- Tajaajila keennaan/keennituun beekumsa kan qabu/du fakaata/tti  6.----- Tajaajila keennaan/keennituun nama simata/tti  7.----- Dhaabbani fayyaa qulqullu dha  8.----- Qorichi gahaan jira  9.----- Tajaajilli barbaadamu asuma qofatti argama  10.----- Tajaajilli keennamu gaarii waan ta’ef  11.----- Tajaajila argachuuf waan hintursisneef  12.----- Ogeesoota waanan beekuf  13.----- Yeroo hunda asin dhufa  14.----- Hiriyooni yokin firri koo nagorsani  15.----- Tajaajilli keennamu iccitti dhunfaa eega  16.----- Sababa addaa hinqabu  Kan biroo(ibsi)-------------------------------88  Hinbeeku---------------------------------------99 | | | | | | | | | |  |  |
| 415 | Yeroo gosa karoora maatii amma fayyadamaa jirtu fudhatte karoora maatii ilaalchisee gorsa argateeraa? | 1.-----------Lakki  2.-----------Eeyyee  Hinyaadadhu-------------------------------------77 | | | | | | | | | |  |  |
| 416 | Gaaffin 415 eeyyee yoo ta’e, gorsa keennaan gosa filatte fi amma fayyadamaa jirtuun walqabatee mata duree /dhimma kam irratti odeeffanno sif keenne?  (Deebiin tokko ol ta’u danda’a) | 1.--- Bu’a qabeesummaa isaa  2.--- Faayidaa fi midhaa isaa  3.---Rakko cinaa yokin rakkolee gosa karoora maatiin walqabatan fi rakkoleen cinaa yoo qunnaman maal godhamuu akka qabu  4.--- Gosa filatte akkamitti fayyadamu akka qabdu/qajeelfamoota  5.--- Yoom deebi’uu akka qabdu  Kan biroo(ibsi)--------------------------------88  Hinyaadadhu-----------------------------------77 | | | | | | | | | |  |  |
| 417 | Gosni karoora maatii amma fayyadamaa jirtu ulfa ittisuuf hammam bu’aaqabeesa jatte yaadda? | 1.---- Baayyee bu’aaqabeessa  2.---- Bu’aaqabeessa  3.---- Hamma ta’e bu’aaqabeessa  4.---- Bu’aaqabeessa miti  Hinbeeku---------------------------------------99 | | | | | | | | | |  |  |
| 418 | Gosa karoora maatii ittifayyadamaa jirtu haala kamin argatte? | 1.----- Bilisaan  2.----- Kaffaltiin | | | | | | | | | |  |  |
| 419 | Ati yokin abbaan warraa kee gosa karoora maatii amma fayyadamaa jirtu argachuuf rakkattanirtuu? | 1.-----------Lakki  2.-----------Eeyyee  Hinyaadadhu-------------------------------------77 | | | | | | | | | | 1, 77 gara 421 |  |
| 420 | Gaaffin 419 eeyyee yoo ta’e, rakkolee maaltu si qunname? | 1.--- Dhaabbanni tajaajila itti argadhe fagoo dha ykn deemuuf hinmijatu  2.--- Bitachuuf gatii guddaa gaafata  3.--- Hojjetoota dhaabbatichaatiin ilaalamuun/ tajaajilamuun rakkisaa dha  4.--- Dhiyeesi karoora maatii cicitaa dha yokin hinargamu  5.--- Mormiin maatii yokin hawaasaa irraa naqunnamee ture  6.---- Dhibbaa amantaa  7.---- Rakkon geejibaa jira  8.---- Hanqinni yeroo jira  Kan biroo (ibsi)-----------------------------------88 | | | | | | | | | |  |  |
| 421 | Gosa karoora maatii amma fayyadamaa jirtu dhaabbata fayyaa kam irraa yoo argatte filatta?  (Deebiin tokko ol ta’u danda’a) | 1.--------- Keellaa Fayyaa  2.--------- Buufata Fayyaa  3.---------- Hospitaala Aanaa  4.---------- Hospitaala Godinaa  5.---------- Kilinika dhunfaa  6.---------- Kuusaa qoricha dhunfaa  7. ---------- Mana qorichaa  8.----------- Hospitaala dhunfaa  Kan biroo(ibsi)------------------------------------88 | | | | | | | | | |  |  |
| 422 | Gosni karoora maatii kan biraa ittifayyadamuuf filattu jiraa? | 1.-----------Lakki  2.-----------Eeyyee  Hinbeeku --------------------------------------99 | | | | | | | | | | 1, 99 gara 424 |  |
| 423 | Gaaffin 422 eeyyee yoo ta’e, gosni kuun maal ta’a? | 1.---- Dhaabbi kan dubartootaa (Tubal ligation)  2.---- Dhaabbi kan dhiraa (Vasectomy)  3.---- Kiniina  4.---- Gadaameesa keessa kan taa’u (IUCD)  5.---- Hirree harkaa jala kan galu (Implants)  6.---- Marfee ji’a sadin keennamu  7.---- Koondoomii  8.----Gadaameesa keesa kan kaawamu (Diaphragm, Foam, Jelly)  9.----Guyyota lakaa’un fayyadamu (Rhythm/Calendar method)  10.----Sanyii dhiraa nafa dubartii alatti dhangalaasuu (Withdrawal Method)  11.----Harma yeroo dheeraaf hosiisuu  Kanbiroo(ibsi)----------------------------------88 | | | | | | | | | |  |  |
| 424 | Ji’ottan 12 darban keessa hojjetoota ekisteenshinii fayyaa/ hojjetoota feedhii fayyaa hawaasaan yokin kan birootiin ilaalamtee waa’ee karoora maatii sidubbisaniru? | 1.----------------------------------------- Lakki  2.-----------------------------------------Eeyyee  Hinyaadadhu----------------------------77 | | | | | | | | | |  |  |
| 425 | Ji’ottan 12 darban keessa tajaajila karoora maatiif dhaabbilee fayyaa deemtee jirtaa? | 1.----------------------------------------- Lakki  2.-----------------------------------------Eeyyee | | | | | | | | | |  |  |
| 426 | Gosa karoora maatii argachuuf eenyuutu murteesse? | 1.---------Baayyinaan deebiistu gaaffi kanaa  2.-------- Baayyinaan abbaawarraan  3.-------- Murtee waliini  4.-------- Nama biraatiin  Kan biroo(ibsi)----------------------------------88 | | | | | | | | | |  |  |
| 427 | Dubartootni gosa karoora maatii kamiyyu kan hinfayyadamne ati beektu jirani? | 1.----------------------------------------- Lakki  2.-----------------------------------------Eeyyee  Hinbeeku---------------------------------99 | | | | | | | | | | 2, 99 gara 429 |  |
| 428 | Gaaffin 427 eeyyee yoo ta’e, yaada keetiin/isaan irraa yoo dhageesse/sababni gosa karoora maatii itti hinfayyadamne maalii? | 1.---Dhaabbanni tajaajila keennu fagoo dha  2.--- Rakkina geejibaa yokin deemuuf hinmijatu  3.--- Ijoolee dabalachuu barbaadu  4.--- Bitachuu hindanda’an  5.--- Rakkolee cinaa/wlaxaxaa sadaachuun  6.--- Simannaan tajaajilaa gaarii ta’u dhisuu/qulqullina kunuunsa gadi anaa ta’e  7.--- Tajaajila argachuuf yeroo dheeraa turu  8.--- Hubanno dhabinsa mala/gosa karoora maatii biroo irratti  9.--- Mormii abbaawarraa/nama walqunnamtii saalaa walin qaban irraa  10.--- Mormii maatii yokin haawaasa irraa qunnameen  11.--- Dhibbaa amantaa  12.--- Rakkon geejibaa jira  Kanbiroo(ibsi)----------------------------------88 | | | | | | | | | |  |  |
| 429 | Waa’ee gosa karoora maatii yeroo dheeraa hirree harkaa jala galu dhageesse beektaa? | Kan hirree harkaa jala galu | 1. Lakki | | | | | 2.Eeyye | | | | 1 lamaanifu gara 450 |  |
|  |  | Kan gadaameessa keessa taa’u (IUCD) | 1. Lakki | | | | | 2.Eeyye | | | |  |  |
| 430 | Gaaffin 429 eeyyee yoo ta’e, waa’ee karoora maatii hirree harka jala galu, gadaameessa keessa taa’u (IUCD) yokin lamaanu eenyu irraa dhageesse? (maddi odeeffanno keetii maalii)? | 1.------ HEF/HFFH(Hojet.Feedhi Fayyaa Hawaasaa)  2.------ Ogeesoota Fayyaa  3.------ Abbaawarraa/nama walqunnamti saalaa walin qaban irraa  4.------ Raadiyoo  5.------ TV  6.------ Barrefamoota adda addaa(gaazeexaa, kitaaba, barulee)  7.------ Miseensoota maatii irraa  8.------ Ollaa irraa  9.------ Hiriyoota irraa  Kanbiroo(ibsi)----------------------------------88 | | | | | | | | | |  |  |
| 431 | Kanaan dura karoora maatii hirree harkaa jala galu kan waggaa sadi fayyadamtee beektaa? | 1.----------------------------------------- Lakki  2.-----------------------------------------Eeyyee | | | | | | | | | | 1 gara 437 |  |
| 432 | Gaaffin 431 eeyyee yoo ta’e,  Eenyuun sif gale? | 1.--------- HEF(Hojjet. Ekist. Fayyaa) tiin  2.--------- Ogeesoota fayyaa tiin | | | | | | | | | |  |  |
| 433 | Yeroo meeqaaf karoora maatii hirree harka jala galu fayyadamte? | Ji’a-----------------/_____/______/  Waggaa -----------/_____/_____//______/_____/  Hinbeeku-------------------------------------------99 | | | | | | | | | |  |  |
| 434 | Dhaabbata fayyaa kamitti sifgale? | 1.--------- Keellaa Fayyaa  2.--------- Buufata Fayyaa  3.---------- Hospitaala Mootummaa  4.---------- Kilinika dhunfaa  5.---------- Hospitaala dhunfaa  6.---------- Dhaabbata miti-mootummaa  Kan biroo(ibsi)------------------------------------88  Hinbeeku-------------------------------------------99 | | | | | | | | | |  |  |
| 435 | Hanga ammaa karoora maatii hirree harka jala galu fayyadamaa jirtaa? | 1.----------------------------------------- Lakki  2.-----------------------------------------Eeyyee | | | | | | | | | | 2 gara 438 |  |
| 436 | Gaaffin 431 lakki yoo ta’e,  Maaliif bahe? | 1.-----Ijoolee godhachuun barbaade  2.----- Filanno abbaawarraan  3.----- Dhibbaa miseensoota maatii irraa  4.----- Dhibbaa amantaa  5.----- Rakko cinaa/walxaxaa sadaachuun  6.----- Sababa oloola ummata irraa dhagaheen  7.----- Gosnii karoora maatii kuun hojjechuu dhabu irraa  8.---- Yeroon ittisa isaa waan dhumeef  Kan biroo(ibsi)------------------------------------88 | | | | | | | | | |  |  |
| 437 | Ati yoo karoora maatii hirree harkaa jala galu hinfayyadamne sababni kee maali? | 1.--- Dhaabbanni tajaajila keennu fagoo dha  2.--- Rakko geejibaa yokin deemuuf hinmijatu  3.--- Bitachuuf gatii guddaa gaafata  4.---Simannaan tajaajila keennotaa gaarii miti/ tajaajilamuuf yeroo dheeraa eeggachuu  5.--- Dhiyeesi karoora maatii cicitaadha yokin hinargamu  6.---Hubanno dhabu  7.----Rakkolee cinaa sodaachuun  8.---- Dhibbaan maatii yokin hawaasaa naqunname  9.---- Ilaalchoota amantaa  10.---- Hanqinni yeroo jira  11.---- Sababa oloola ummata biroo iraa dhagaheen  Kan biroo (ibsi)-----------------------------------88 | | | | | | | | | |  |  |
| 438 | Karoora maatii hirree harka jala galu ilaalchisee odeeffanno/beekumsi ati qabdu isa kami? | “Norplant” (muka abbaa jahaa) kan jadhamu waggaa shaniif /7 ulfa ittisu danda’a | | | | | 1.Lakki  2.Eeyye | | | | |  |  |
|  |  | “Jaddel” (muka abbaa lamaa) kan jadhamu waggaa shaniif ulfa ittisu danda’a | | | | | 1.Lakki  2.Eeyye | | | | |  |  |
|  |  | Impilaanoon (muka abbaa tokko) waggaa sadiif ulfa ittisuu danda’a | | | | | 1.Lakki  2.Eeyye | | | | |  |  |
|  |  | “Implants” Kan hirree harkaa jala galan yeroo galan fi bahan opireeshinii xiqaa akka barbaadan | | | | | 1.Lakki  2.Eeyye | | | | |  |  |
|  |  | “Implants” Kan hirree harkaa jala galan yeroo bahan dafani ulfaa’un akka danda’amu | | | | | 1.Lakki  2.Eeyye | | | | |  |  |
| 439 | Karoori maatii hirree harkaa jala galu implaanooni dhaabbata kamitti akka argamu fi akka keennamu beektaa? | 1.---------------------------------Lakki  2.---------------------------------Eeyyee | | | | | | | | | |  |  |
| 440 | Gaafiin 439 eeyyee yoo ta’e, dubartiin implaanooni fayyadamu barbaadde tajaajila eessaa argatti? | 1.--------- Keellaa Fayyaa HEFtiin  2.--------- Buufata Fayyaa  3.---------- Kilinika dhunfaa  4.---------- Hospitaala mootummaa  5.---------- Hospitaala dhunfaa  Kan biroo (ibsi)---------------------88 | | | | | | | | | |  |  |
| 441 | Karoora maatii gadaameesa keessa taa’u “IUCD”(waggaa 10 ol tajaajilu) fayyadamtee beektaa? | 1.---------------------------------Lakki  2.---------------------------------Eeyyee | | | | | | | | | |  |  |
| 442 | Gaafiin 441 eeyyee yoo ta’e, eenyuutu sif galche? | 1.----HEF(Hojjet. Ekisten. Fayyatiin)  2.----Ogeesoota Fayyaatiin | | | | | | | | | |  |  |
| 443 | Karoora maatii gadaameessa keessa taa’u “IUCD” yeroo meeqaaf fayyadamte? | Ji’a--------------------/_____/______/  Waggaa--------/___/____//____/____/ | | | | | | | | | |  |  |
| 444 | Dhaabbata fayyaa kam keessatti sif gale? | 1.--------- Keellaa Fayyaa  2.--------- Buufata Fayyaa  3.---------- Hospitaala mootummaa  4.---------- Kilinika dhunfaa  5.---------- Hospitaala dhunfaa  6.-------- Dhaabbata miti-mootummaa  Kan biroo (ibsi)---------------------88  Hinbeeku----------------------------99 | | | | | | | | | |  |  |
| 445 | Amma karoora maatii gadaameessa keessa taa’u “IUCD” fayyadamaa jirtaa? | 1.---------------------------------Lakki  2.---------------------------------Eeyyee | | | | | | | | | |  |  |
| 446 | Gaafiin 445 lakki yoo ta’e, maaliif bahe? | 1.---Ijoolee dabalachuuf  2.---Abbaan warraa mormee  3.--- Dhibbaa maatii irraa  4.--- Dhibbaa amantaa  5.--- Sodaa rakko cinaa/walxaxaa irraa  6.--- Sababa oloola ummata biraa irraa dhagaheen  7.--- hojjechuu dhabu irraa  8.---Yeroon isaa dhumee  Kan biroo (ibsi)---------------------88 | | | | | | | | | |  |  |
| 447 | Yoo karoora maatii gadaameessa keessa taa’u “IUCD” hinfayyadamne sababni kee maali? | 1.--- Dhaabbanni tajaajila keennu fagoo dha  2.--- Rakko geejibaa yokin deemuuf hinmijatu  3.--- Bitachuuf gatii guddaa gaafata  4.---Simannaan tajaajila keennotaa gaarii miti/ tajaajilamuuf yeroo dheeraa eeggachuu  5.--- Dhiyeesi karoora maatii cicitaadha yokin hinargamu  6.---Hubanno dhabu  7.----Rakkolee cinaa sodaachuun  8.---- Dhibbaa maatii yokin hawaasaa naqunname  9.---- Ilaalchoota amantaa  10.---- Hanqinni yeroo jira  11.---- Sababa oloola ummata biroo iraa dhagaheen  Kan biroo (ibsi)--------------------88 | | | | | | | | | |  |  |
| 448 | Karoora maatii gadaameesa keessa taa’u “IUCD” ilaalchisee odeeffannoon/ beekumsi ati qabdu isa kami? | Karoora maatii gadaameesa keessa taa’u “IUCD” waggaa 10 ol ulfa ittisa | | | | | 1.Lakki  2.Eeyye | | | | |  |  |
|  |  | Karoori maatii gadaameesa keessa taa’u “IUCD” dubartii dhukkuba naf-saalaan qabamuuf saaxilamteef mija’aa miti | | | | | 1.Lakki  2.Eeyye | | | | |  |  |
|  |  | Karoori maatii gadaameesa keessa taa’u “IUCD” feedhi yokin walqunnamtii saalaa irratti dhibbaa hinfidu | | | | | 1.Lakki  2.Eeyye | | | | |  |  |
|  |  | Yoo karoori maatii gadaameesa keessa taa’u “IUCD” bahe dubartiin daftee ulfa’u dandeessi | | | | | 1.Lakki  2.Eeyye | | | | |  |  |
|  |  | Karoori maatii gadaameesa keessa taa’u “IUCD” xannacha/kaansari hinfidu | | | | | 1.Lakki  2.Eeyye | | | | |  |  |
| 449 | Karoori maatii gadaameesa keessa taa’u “IUCD” dhaabbata kamitti akka argamu fi akka keennamu beektaa? | 1.---------------------------------Lakki  2.---------------------------------Eeyyee | | | | | | | | | |  |  |
| 450 | Gaafiin 449 eeyyee yoo ta’e, dubartiin karoora maatii gadaameesa keessa taa’u “IUCD” fayyadamu barbaadde tajaajila eessaa argatti? | 1.--------- Keellaa Fayyaa HEFtiin  2.--------- Buufata Fayyaa  3.---------- Kilinika dhunfaa  4.---------- Hospitaala mootummaa  5.---------- Hospitaala dhunfaa  Kan biroo (ibsi)---------------------88 | | | | | | | | | |  |  |
| 451 | Gara fuladuraaf karoora maatii fayyadamuuf karoora qabdaa? | 1.---------------------------------Lakki  2.---------------------------------Eeyyee | | | | | | | | | |  |  |
| 452 | Gaafiin 451 eeyyee yoo ta’e, kaayyoo maaliif? | 1.------------- da’umsa addaan fageessuf  2.------------- da’umsa dhaabuuf  Kan biroo (ibsi)---------------------88 | | | | | | | | | |  |  |
| 453 | Ji’otan 12 darban (ogeesoota fayyaa malee) waa’ee karoora maatii namni waliin mari’atte jiraa? | 1.---------------------------------Lakki  2.---------------------------------Eeyyee | | | | | | | | | |  |  |
| 454 | Gaafiin 453 eeyyee yoo ta’e, eenyu waliin mari’atte? | 1.----- Abbaa warraa  2.----- Miseensoota maatii  3.----- Hiriyoota  4.----- Ollaa  Kan biroo (ibsi)------------------------------88 | | | | | | | | | |  |  |
| 455 | Maatii kee keessatti kunuunsa fayyaa ofi argachuuf jacha xumuraa kan jedhu eenyuu? | 1.------- Gaafatamtu qofa  2.------- Gaafatamtu fi Abbaawarraa waliin  3.------- Gaafatamtu fi nama tokko  4.------- Abbaa warraa  5.------- Mana keessaa nama tokko | | | | | | | | | |  |  |

Gaaffilee koo xumureera galatoomi.

## Annex II. Qualitative tools for Assessment of Family Planning Services Utilization

Information Sheet and Consent Form

Good morning/afternoon/evening. My name is _________________________ I represent the research team of Ethiopia Public Health Association. We are currently doing a study about utilization of family planning methods in Western part Oromia Regional State. As a part of this study, we are interviewing family planning service providers (health extension workers (HEWs) and health professionals. I will only ask you very simple, brief & general questions in relation to family planning service. The result of this interview will help in better understanding of family planning service delivery and to improve family planning utilization.

Your participation in this study is completely on voluntary bases means you can refuse to participate in the study entirely or you can refuse to answer any question. There are no anticipated problems you encounter because of your participation in this study. The interview will last approximately 40 -45 minutes. I would like to inform you that the information we collect from you will not be shown to anyone outside of this project.

I would like to kindly inform you that our interview will be tape recorded in order not to miss points raised. If you have a mobile phone please switched it off or makes it silent until the end of the session.

May I proceed with the questions? Yes --------------- No -----------------

Name of the health Post ________________________

Woreda: _________________________________kebele: _____________

Zone_________________________________________

Interview Code: _______________

Name of Interviewer: ________________Date___/____/_____ Signature________

**Focus group discussion (FGD) guide with Family Planning Service Providers**

1. **General questions**
   1. Sex: **___________** Age: **__________**year
   2. Would you tell me your profession and academic level?
   3. For how long you have been working as a family planning service provider (probe: Total experience and at this facility)
   4. From your experience, would you describe the profile of women seeking family planning service from this facility?(probe: age, educational status, marital status, religion, residence…)
2. **Family planning service delivery with emphasis of LAFP methods (Availability, method mix and utilization)**
   1. Would you please mention available family planning methods in this facility and problems

in relation to availability of various FP methods and related supplies?

- 1. How do you see the women’s current choice and utilization of family planning methods?
  2. Are LAFP methods (Implants and IUCD) known, easy for access and being properly chosen and utilized by the women?
  3. If LAFP methods (Implants and IUCD) are not being chosen and used by women, what do you think about the major reasons? Misconception women have concerning LAFP methods?
  4. Common complaints raised by women who were/are using implants/ implanon? And reasons for discontinuation?
  5. Common complaints raised by women who were/are using IUCD? And reasons for discontinuation?
  6. Would you mention the barriers of providing LAFP services and factors influencing LAFP methods utilization?

**3. Training andNeed for additional training**

3.1. Have you got in-service trainings on LAFP methods service provision? If yes, Probe:

- Specifically on which training and on which method? By whom?
- The importance and relevance of the skills and knowledge acquired in that training?
- What was lacking in the training?
  1. How do you see your skill of providing implants/ implanon? And IUCD?
  2. What training (additional) do you need on LAFP to provide quality LAFP service?

5. Anything you want to say or suggest to improving the quality of FP service delivery?

**In-depth interview guide with the Health Extension Workers (HEWs)**

- - - 1. **General questions**
  1. . Age of respondent: ___________ year
  2. . For how long you have been working as a health extension worker? (Total experience and experience at this health post/kebele)
  3. .Please tell me family planning services you are providing in your community or at your HP?

1. **Availability and utilization of FP service particularly LAFP methods**.
   1. Which family planning methods are available here in your health post?
   2. How do you see women’s current choice and utilization of family planning methods?The most commonpreferred and used methods/
   3. Do you provide LAFP methods? **if yes, probe**

- Which type of LAFP methods (Implants, IUCD)
- Are these types of method known by women, accessible and properly chosen and used by Women?

If the health post **does not provide** LAFP methods like implanon

- What do you think about the major reasons for not providing LAFP methods like implanon?
  1. How do you see your family planning service counseling? (probe: method choice and side effects, privacy, confidentiality)
  2. What are the major reasons/concerns women mention for not choosing and using LAFP such as implanon and IUCD? Misconception women have concerning LAFP methods?
  3. Common complaints reported by women who were/are using LAFP methods? And reasons for discontinuation of LAFP methods?

1. **Training and Need for additional training?**
   1. Have you got in-service trainings on FP service provision? If yes, probe:

- Specifically on which training and on which method? By whom?
- The importance and relevance of the skills and knowledge acquired in that training?
- What was lacking in the training?
  1. How do you see your skill of providing LAFP methods specifically implanon insertion and removal?
  2. What kind of training (additional) do you need to improve your skill of providing LAFP methods?

1. **Challenges and Barriers**
   1. Would you mention the challenges or barriers of providing family planning services specifically LAFP methods in your community?
   2. What do you suggest to solve these challenges/problems?

Information Sheet and Consent Form

Good morning/afternoon/evening. My name is _________________________ I represent the research team of Ethiopia Public Health Association (EPHA). We are currently doing a study about utilization of family planning methods in Western part Oromia Regional State. As a part of this study, we are interviewing the headof health facilities, Woreda and Zonal family health unit coordinators. I will only ask you very simple, brief & general questions in relation to family planning service. The result of this interview will help in better understanding of family planning service delivery and to improve family planning utilization.

Your participation in this study is completely on voluntary bases means you can refuse to participate in the study entirely or you can refuse to answer any question. There are no anticipated problems you encounter because of your participation in this study. The interview will last approximately 40 -45 minutes. I would like to inform you that the information we collect from you will not be shown to anyone outside of this project.

I would like to kindly inform you that our interview will be tape recorded in order not to miss points raised. If you have a mobile phone please switched it off or makes it silent until the end of the session.

May I proceed with the questions? Yes --------------- No -----------------

Name of the health facility ________________________

Woreda _______________________________________

Zone_________________________________________

Interview Code: _______________

Name of Interviewer: ________________Date___/____/_____ Signature________

**In-depth interview guidewith Head of Health Facility**

1. **General questions**
   1. Sex: **___________**
   2. Would you tell me your profession and academic level?
   3. Your work experience in managerial positions (probe: Total and at this facility)

**2. Family planning service delivery with emphasis of LAFP methods (Availability, method mix and utilization)**

2.1.Do you think that the health facilityis adequately organized and supplied to provide proper FP services?Probe:

- Does the facility hasseparate room for provision of FP methods?
- Does the facility has trained and competent FP service provider?
- What about the availability of various contraceptive methods particularly Implants and IUCD and stock out of these methods?
  1. How do you notice the involvement of HEWs in providing wider range FP services?andLAFP methods? Their skill of providing Implanon and IUCD insertion and removal)
  2. Would you mention the barriers of providing LAFP services and factors influencing LAFP methods utilization?
  3. Are there any FP/RH program interventions implemented in the catchment area?

**3. Training andNeed for additional training**

3.1. Have you got in-service training on RH/FP leadership? If yes, Probe:

- The importance and relevance of the skills and knowledge acquired in that training?
- What was lacking in the training?

3.2. What kind of training (additional) do family planning service providers need on LAFP methods?

**4.** Anything you want to say or suggest to improving the quality of FP service delivery?

**In-depth interview guide with Zonal Family Health Unit Coordinator**

1. **General**
   1. Sex: **___________**
   2. Would you tell me your profession and academic level?
   3. Your work experience in managerial positions (probe: Total and at this office)
2. **Family planning service provision and utilization in the zone**
   1. How do you see family planning (both short term and long term) services utilization and demand in the zone? (Which contraceptive method is/are mostly used by women, met and unmet need for family planning of the zone
   2. Would you please describe availability and provision of LAFP methods in your zone?
   3. How do you see the quality of LAFP services provision? (Proper insertion and removal of LAFP methods)
   4. Would you mention the barriers of providing LAFP services and factors influencing LAFP methods utilization?
3. **Training, evidences and best practices on FP services and training need**
   1. Is there any training given regarding FP services provision? (Check for Implanon and IUCD insertion and removal training and number of HEWs, supervisors, nurses and /or midwives trained
   2. Is there any training given for leaders/ heads on FP/RH leadership? if yes, number of leaders trained
   3. Are there evidences/researches done on FP service provision and utilization and existing evidence indicating the problem areas that need to be addressed and best practices to be expanded?
   4. Is there training institutions under the zone which train Midwives, clinical nurses, health extension workers? if yes, how do you see the linkage between the zone and the training institution particularly in family planning service provision?
   5. What do you **say and suggest**to improve family planning service provision and to increase family planning service utilization/coverage?
4. **E- Healthusing mobile phone technology (Awareness, experience (if any), preparation, enabling conditions and anticipated challenges)**
   1. Have your heard about the national E-health initiatives using mobile phone technology?
   2. Is E- health-technology introduced and set in place? If yes, share us your experiences?
   3. What preparations the zone is doing to implement this innovative strategy?(What activities the zone is carrying out in this regard, types of health care included, messages developed, any supporting documents prepared for the users, etc.)
   4. What are the enabling conditions in your zone for successful implementation of E-health using mobile phone technology?
   5. What are/will be the challenges of implementing E-health using mobile phone technology in your zone?

**In-depth interview guide with Woreda MCH/ Family Health Unit Coordinator**

1. **General questions**
   1. Sex: **___________**
   2. Would you tell me your profession and academic level?
   3. Your work experience in managerial positions (probe: Total and at this office)

**2. Family planning service provision and utilization in the Woreda**

- 1. How do you see family planning (both short term and long term) services utilization and demand in the Woreda? (Which contraceptive method is/are mostly used by women, met and unmet need for family planning of the zone
  2. Would you please describe availability and provision of LAFP methods in your Woreda?
  3. How do you see the quality of LAFP services provision? (Proper insertion and removal of LAFP methods)
  4. Would you mention the barriers of providing LAFP services and factors influencing LAFP methods utilization?

**3. Training, evidences and best practices on FP services and training need**

- 1. Is there any training given regarding FP services provision? (Check for Implanon and IUCD insertion and removal training and number of HEWs, supervisors, nurses and /or midwives trained
  2. Is there any training given for leaders/ heads on FP/RH leadership? if yes, number of leaders trained
  3. Are there evidences/researches done on FP service provision and utilization and existing evidence indicating the problem areas that need to be addressed and best practices to be expanded?
  4. How do you see the linkage between the Woreda and the training institution which train Midwives, clinical nurses, health extension workers particularly in family planning service provision?
  5. What do you **say and suggest** to improve family planning service provision and to increase family planning service utilization/coverage in your Woreda?

**5. E- Health using mobile phone technology (Awareness, experience (if any), preparation, enabling conditions and anticipated challenges)**

- 1. Have you heard about the national E-health initiatives using mobile phone technology?
  2. Is E- health-technology introduced and set in place? If yes, share us your experiences?
  3. What preparations the Woreda is doing to implement this innovative strategy? (What activities the Woreda is carrying out in this regard, types of health care included, messages developed, any supporting documents prepared for the users, etc.)
  4. What are the enabling conditions in your Woreda for successful implementation of E-health using mobile phone technology?
  5. What are/will be the challenges of implementing E-health using mobile phone technology in your Woreda?

Information Sheet and Consent Form

Good morning/afternoon/evening. My name is _________________________ I represent the research team of Ethiopia Public Health Association (EPHA). We are currently doing a study about utilization of family planning methods in Western part Oromia Regional State. As a part of this study, we are interviewing the headof Midwifery or Clinical Nursing training colleges/schools and instructors who thought reproductive health/family planning. I will only ask you very simple, brief & general questions in relation to family planning service. The result of this interview will help in better understanding of the curriculum used for training Midwive and clinical nurse students and the training approach of the college/school.

Your participation in this study is completely on voluntary bases means you can refuse to participate in the study entirely or you can refuse to answer any question. There are no anticipated problems you encounter because of your participation in this study. The interview will last approximately 40 -45 minutes. I would like to inform you that the information we collect from you will not be shown to anyone outside of this project.

I would like to kindly inform you that our interview will be tape recorded in order not to miss points raised. If you have a mobile phone please switched it off or makes it silent until the end of the session.

May I proceed with the questions? Yes --------------- No -----------------

Name of the College/School ________________________

Woreda _______________________________________

Zone_________________________________________

Interview Code: _______________

Name of Interviewer: ________________Date___/____/_____ Signature________

**In-depth interview guide with the dean of Midwifery/ Nursing training institution**

1. **General Questions**
   1. Name of the college/ School __________________________
   2. Year of its establishment _____________________________
   3. Type of training program of the college? (Generic, Upgrading, …by level....)
   4. Number of graduates by profession and level graduated with in the last five years?
2. **Family planning/Reproductive health training**
   1. How do you see thecurriculum used for training midwive and clinical nurse trainee particularly reproductive health /family planning course/topic?

- Briefly tell us how many credit hours does it have? (theoretical + practical sessions), training material/aids used for the training
- What about the training session on LAFP (theoretical, practical session it has, having training materials like Implanon, IUCD and other supplies)
  1. Is special training on LAFP like pre-service training being organized and provided to graduating class students of yourcollege/school? If yes
- By whom the training is organized? When was it started?Is it currently provided?
  1. How many graduates have been graduated with proper LAFP services provision skills? Any follow up done after graduation, and etc.
  2. Is there pre-service training given to graduating class students on other components of reproductive health?
- When it stated? On what topics/issues?and in collaboration with which organization your college provided the training?

1. Linkage with Health service delivery points(Hospitals, health centers)
   - 1. Does the college established linkage with the nearby health facilities? If yes,  list names of HCs or Hospitals
     2. Type of supports given to HCs/HPs and type of support the college gains from health facilities? Material provision, practical sessions/attachments done in HCs/HPs. Other points to add
2. **Human resources**
   1. Do you have enough human resources?
   2. Briefly tell us about the number and qualification of the trainers, particularly FP services provision trainers (LAFP)?
   3. Is capacity building training on family planning service provision /LAFP provided to instructors? If yes, on what topics, and how many instructors were trained

**Focus group discussion (FGD) guide with Midwifery /clinical nursing training College instructors**

- 1. How do you evaluate the curriculum of Midwifery/ Nursing profession with regard to training RH/FP issues? Is FP considered as a course or topic in the curriculum, with how many credit hour/contact hour, what about the practical session.
  2. What about training materials like FP supplies, equipments,
  3. Do you think that students have acquired the basic knowledge and skill of FP service provision at the time of graduation?
  4. What difficulties or deficiencies students demonstrated during practical attachment on provision of family planning services?
  5. How do you see the importance of pre- service training on RH/FP for graduating class students? What values the training added?

Information Sheet and Consent Form

Good morning/afternoon/evening. My name is _________________________ I represent the research team of Ethiopia Public Health Association (EPHA). We are currently doing a study about utilization of family planning methods in Western part Oromia Regional State. As a part of this study, we are interviewing women of child bearing agein group. I will only ask you very simple, brief & general questions in relation to family planning service. The result of this interview will help in better understanding of the family planning service utilization and associated factors.

There are no anticipated problems you encounter because of your participation in this study. The discussion will last approximately 40-45 minutes. I would like to inform you that the information we collect from you will not be shown to anyone outside of this project, used for research purpose only.

There are no wrong or right answers.All comments, both positive and negative to the point of discussion are welcomed.Please feel free to express your opinions. We would like to have many points of view.We will audiotape all your comments and opinions so that we could not miss any of your ideaswhile trying to take notes.

If you have a mobile phone please switched it off or makes it silent until the end of the session.

May I proceed with the questions? Yes --------------- No -----------------

Woreda _______________________________________

Zone_________________________________________

Interview Code: _______________

Name of Interviewer: ________________Date___/____/_____ Signature________

**Focus group discussion (FGD) guide witheligible women for family planning services**

1. Do people in your community know ways to avoid becoming pregnant?
2. How do you see the benefits/ importance of family planning services?
3. What are the enabling conditions for using family planning services in your village?
4. How do you see the family planning service utilization in your locality? Who were/are the users, accessibility of various contraceptive methods (Implanon and IUCD)
5. Why do people in your community not using modern contraceptives? What are the barriers of using modern family planning methods? Cultural, economical, religious, and other factors, what about the role of Male partner for using FP methods?
6. How do you see the family planning service delivery? (Its accessibility, service providers approach and interaction, quality of service in general)
7. What is your view regarding HEWs providing LAFP services (Implanon, and IUCD insertion and removal) in your Kebele?
8. What do you think should be done to improve contraceptive use in your community?

Thank you so much for coming to this session.
